# Impacts of Epigenetic Processes on the Health and Productivity of Livestock

**DOI:** 10.3389/fgene.2020.613636

**Published:** 2021-02-23

**Authors:** Mengqi Wang, Eveline M. Ibeagha-Awemu

**Affiliations:** ^1^Agriculture and Agri-Food Canada, Sherbrooke Research and Development Centre, Sherbrooke, QC, Canada; ^2^Department of Animal Science, Laval University, Quebec, QC, Canada

**Keywords:** DNA methylation and histone modification, epigenetics biomarker, application of epigenetics data, growth and development, livestock, productivity, health and disease

## Abstract

The dynamic changes in the epigenome resulting from the intricate interactions of genetic and environmental factors play crucial roles in individual growth and development. Numerous studies in plants, rodents, and humans have provided evidence of the regulatory roles of epigenetic processes in health and disease. There is increasing pressure to increase livestock production in light of increasing food needs of an expanding human population and environment challenges, but there is limited related epigenetic data on livestock to complement genomic information and support advances in improvement breeding and health management. This review examines the recent discoveries on epigenetic processes due to DNA methylation, histone modification, and chromatin remodeling and their impacts on health and production traits in farm animals, including bovine, swine, sheep, goat, and poultry species. Most of the reports focused on epigenome profiling at the genome-wide or specific genic regions in response to developmental processes, environmental stressors, nutrition, and disease pathogens. The bulk of available data mainly characterized the epigenetic markers in tissues/organs or in relation to traits and detection of epigenetic regulatory mechanisms underlying livestock phenotype diversity. However, available data is inadequate to support gainful exploitation of epigenetic processes for improved animal health and productivity management. Increased research effort, which is vital to elucidate how epigenetic mechanisms affect the health and productivity of livestock, is currently limited due to several factors including lack of adequate analytical tools. In this review, we (1) summarize available evidence of the impacts of epigenetic processes on livestock production and health traits, (2) discuss the application of epigenetics data in livestock production, and (3) present gaps in livestock epigenetics research. Knowledge of the epigenetic factors influencing livestock health and productivity is vital for the management and improvement of livestock productivity.

## Introduction

Increasing animal food demand by an ever-expanding human population as well as the challenges of global climate change is a clarion call for the sustainable development of the food animal industry, with the expectation of increased supply of high-quality animal proteins with minimal environmental impacts. In response, recent research efforts are geared towards developing different approaches to improve livestock production efficiency, decrease production cost, and develop environmentally friendly livestock production systems (Capper and Bauman, 2013; Scott, 2018). In recent years, the application of modern technologies including advanced sequencing technologies, genotype analysis, and genome profiling has promoted important changes in livestock genetic breeding programs and gains in important livestock traits like milk yield/quality in dairy cattle and goat, meat quality in beef cattle and swine, egg yield/quality in chickens, etc. Continued technological advances have further promoted the implementation of genomic selection in livestock production (Georges et al., 2019; Gurgul et al., 2019). Sequence analysis of livestock genomes uncovered the general molecular and regulatory mechanisms of the coding and non-coding genome underlying production and health traits, which have supported advances in trait improvement (Kamath et al., 2016; Do et al., 2017a; Wara et al., 2019). These factors still fall short of accounting for the optimal level of variation that is required to achieve continued improvements in livestock health and productivity. The epigenome, which responds to internal and external environmental cues, is less explored but contains additional levels of variation that could be exploited for livestock trait improvement.

Epigenetics is defined as the study of heritable molecular modifications responsible for the regulation of genome activities and gene expression, resulting in phenotypic differences without alterations to the basic DNA sequence (Nicoglou and Merlin, 2017; Greally, 2018). Epigenetic processes, which include DNA methylation, histone modification, chromatin remodeling, and non-coding RNA (ncRNA) regulation, regulate gene expression and, thus, play significant roles in genome function and stability (Bird, 2002; Kouzarides, 2007; Morris and Mattick, 2014; Do and Ibeagha-Awemu, 2017). The epigenome, which encompasses these epigenetic processes, is dynamic during the whole lifetime and is significantly associated with the interaction between genetic activities and environmental stimulation (Bernstein et al., 2007; Monk et al., 2019). Sufficient evidence from epigenetics-related studies in humans and animals have demonstrated the distinctive roles of epigenetic mechanisms in various biological processes, such as growth, development, metabolism, and health (Ibeagha-Awemu and Zhao, 2015; Paiva et al., 2019). The occurrence of epigenetic mechanisms with important roles at specific key times of development or pathological conditions may be key to further exploration of the intricacies of diseases. In addition, the awareness and use of epigenetic mechanisms could be advantageous to the understanding of quantitative traits and in achieving advancements in the improvement of livestock productivity and disease resistance (Ibeagha-Awemu and Zhao, 2015; Ibeagha-Awemu and Khatib, 2017; Banta and Richards, 2018; Panzeri and Pospisilik, 2018). The important contribution of epigenetic processes to phenotypic outcome in livestock is beginning to attract attention implying that the impacts of these processes may soon find application in advancing livestock productivity and health (Doherty et al., 2014; Goddard and Whitelaw, 2014; Meirelles et al., 2014; Ibeagha-Awemu and Zhao, 2015; Triantaphyllopoulos et al., 2016; Ibeagha-Awemu and Khatib, 2017; Yakovlev, 2018; Paiva et al., 2019). This review has been categorized into sections that concentrate on discussing the epigenetics processes and impacts on gene regulation; evidence of the impacts of epigenetic regulatory mechanisms underlying productivity and health in different species of livestock animals, such as bovine, swine, sheep, goat, poultry, and other species; application of epigenetics data in livestock production; and research gaps and future perspectives. Evidence of epigenetic impacts on livestock reproduction and epigenetic alterations due to assisted reproduction technologies have been addressed in several recent reviews (Weksberg et al., 2007; Das et al., 2017; Franco, 2017; Khezri et al., 2020; Rivera, 2020; Wang et al., 2020e) and will not be discussed here.

## Epigenetic Processes and Impacts on Gene Regulation

### DNA Methylation

DNA methylation is thus far the most stable and characterized epigenetic modification in most mammalian genomes. DNA methylation principally occurs in the fifth carbon of cytosine residues (addition of a methyl or hydroxymethyl group, denoted as 5mC or 5hmC, respectively) in DNA sequence and mostly at cytosine-phosphate-guanosine (CpG) dinucleotides and to a lesser extent at cytosine-phosphate-adenosine (CpA), cytosine-phosphate-thymine (CpT), and cytosine-phosphate-cytosine (CpC) dinucleotides. The formation of DNA methylation patterns is catalyzed by the activities of a class of enzymes known as DNA methyltransferases (DNMTs). While DNMT3A and DNMT3B are responsible for the establishment of DNA methylation patterns during embryonic development or in response to environmental challenges, DNMT1 maintains DNA methylation during cell division (Edwards et al., 2017; Luo et al., 2018; Schmitz et al., 2019). DNMT3L, which acts as a co-factor of DNMT3A and DNMT3B during *de novo* DNA methylation, is required for mammalian genome imprinting (Hanna and Kelsey, 2014; Basu, 2016; Veland et al., 2019). There are other DNMTs which act in different biological processes. For example, DNMT3C is responsible for the silencing of young retrotransposons (Barau et al., 2016), while DNMT2 plays active roles in RNA methylation and the expression of small ncRNAs (Raddatz et al., 2013; Jeltsch et al., 2017; Zhang et al., 2018b). However, when established DNA methylation is not maintained, the process of passive or active demethylation sets in. Passive demethylation is through the activities of TET (ten–eleven translocation methylcytosine dioxygenases) proteins (e.g., TET1, TET2, and TET3) which mediate the oxidation of 5mC to produce 5-hydroxymethylcytosine (5hmC), 5-formylcytosine (5fC), and 5-carboxylcytosine (5caC; He et al., 2011; Wu and Zhang, 2017). Active demethylation is when replication-dependent dilution of 5hmC, 5fC, and 5caC or thymine DNA glycosylase (TDG)-mediated excision of 5fC and 5caC is coupled with base excision repair (Wu and Zhang, 2017). Other TET–TDG-independent mechanisms are also proposed to mediate active DNA demethylation (Wu and Zhang, 2010, 2014; Bochtler et al., 2017).

The impact of DNA methylation on gene expression is associated with its genome distribution. CpG-rich regions (also known as CpG islands) are frequently distributed in the promoter regions (usually extends to 5′UTR) and usually non-methylated, whereby its abnormal methylation may cause the repression of corresponding transcription and gene silencing (Deaton and Bird, 2011; Smith et al., 2020). About 72% of promoters are within CpG islands and nearly unmethylated, and their activities might be regulated by DNA methylation (Saxonov et al., 2006; Deaton and Bird, 2011). Promoter CpG islands have differential susceptibility to methylation during normal development or during disease progression (e.g., carcinogenesis), which might be influenced by intrinsic sequence properties (Feltus et al., 2006). Most promoter CpG islands (usually unmethylated) escape from *de novo* methylation during all developmental stages, and the activity of promoters with intermediate to high CpG content was negatively correlated with their DNA methylation status (Weber et al., 2007). However, there are still a small number of methylated CpG islands in gene promoters, such as at germline imprinting control regions, or on the inactive X chromosome in female somatic cells (Proudhon et al., 2012). DNA methylation could perturb gene expression activities through direct inhibition of transcription factor (TF) binding or indirect mediation by methyl-binding domain (MBD) proteins that recruit chromatin-modifying activities to methylated DNA (Razin and Kantor, 2005; Zhu et al., 2016; Yin et al., 2017; Greenberg and Bourc’his, 2019). It has been noted that TFs are likely to induce local epigenetic remodeling (Wapinski et al., 2013). DNA methylation in recognition sequences of some TFs was revealed to alter their binding specificity (Zhu et al., 2016). It was reported that DNA methylation of target sequences diminished the binding activity of numerous TFs in the human genome, whereas some TFs of the extended homeodomain family preferred CpG methylated sequences (Yin et al., 2017). The identification of MBDs, such as methyl-CpG binding protein 1 (MeCP1) and methyl-CpG binding protein 2 (MeCP2), revealed that DNA methylation is connected with chromatin structure and gene expression. Except for binding to CpG-rich heterochromatin, some MBDs contain a transcriptional repressor complex that may induce histone deacetylation and chromatin remodeling, contributing to gene silencing (Razin and Kantor, 2005; Greenberg and Bourc’his, 2019).

DNA methylation is also found in the gene body, especially in introns, and is prone to high levels of methylation (Maunakea et al., 2010). DNA methylation in gene body is highly conserved across eukaryotes and has been positively correlated with transcription (Lister et al., 2009; Varley et al., 2013), indicating potential functions other than gene silencing. Two hypotheses underlying the function of DNA methylation in gene bodies have been suggested (Greenberg and Bourc’his, 2019). On the one hand, DNA methylation enriched at exons influences splicing and gene expression (Gelfman et al., 2013; Yang et al., 2014; Shayevitch et al., 2018). DNA methylation was found to facilitate exon exclusion by preventing CCCTC-binding factor (CTCF) binding or contribute to exon inclusion by recruitment of MeCP2 or splicing factors (Shukla et al., 2011; Maunakea et al., 2013; Yearim et al., 2015). However, these mechanisms could only explain a small portion of alternative splicing events. On the other hand, gene body DNA methylation suppresses intragenic promoters consistent with the possible role of DNA methylation as a transcriptional repressor. It was reported that binding of ADD domain to H3K36me3 released the inhibition of DNMT3 enzymes thereby promoting the establishment of *de novo* DNA methylation and, consequently, inhibition of cryptic promoters (Carrozza et al., 2005; Greenberg and Bourc’his, 2019). Indeed, methylation of CpG island in gene body suppressed promoter activity, and altered methylation contributed to the regulation of transcription initiation in a tissue- and cell-type-specific mechanism in mammals (Maunakea et al., 2010). Compared with its well-established repressive function at regulatory elements (such as the promoter region), less is known about DNA methylation regulation and function(s) at intergenic regions. Intergenic regions are mainly populated by regulatory ncRNA genes and other regulatory elements yet to be described, and the DNA methylation at these regions potentially regulates these factors. Downstream regions of genes contain miRNA binding sites, which may interact with DNA methylation and regulate gene expression. For example, the interaction between piwi RNA and DNA methylation is dedicated to silencing transposable elements in the germline (Manakov et al., 2015; Barau et al., 2016).

### Histone Modifications

Histone modification is another important epigenetic mechanism with significant impacts on chromatin regulation and regulation of transcription processes (Adamczyk, 2019). Histones are a family of proteins (H1/H5, H2A, H2B, H3, and H4) that park and order the DNA molecule into structural units called nucleosomes. The N-terminal tails of histones are subjected to various posttranscriptional or posttranslational modifications with more than 100 forms (e.g., lysine acetylation, lysine methylation, ubiquitination, serine/threonine phosphorylation, crotonylation, sumoylation, etc.) with varying effects on transcriptional activities described (Kouzarides, 2007; Zhao and Shilatifard, 2019; Roma-Rodrigues et al., 2020). Histone acetylation and methylation frequently occurs in the lysine of the N-terminal tails resulting from the interaction of associated enzymes or factors (Srivastava et al., 2016). Histone acetyltransferases are responsible for histone acetylation, which play important roles in releasing chromatin structure (histone–DNA interaction) and promoting transcriptional activities, while histone deacetylases cause deacetylation to repress gene expression (Dose et al., 2011; Schmauss, 2017). Similarly, the dynamic changes of histone methylation, which are generally classified into tri-, di-, and monomethylation of lysine residues and the monomethylation of arginine residues, are regulated by histone methyltransferases and demethylases (Ye et al., 2017). The impact of histone methylation on transcriptional activity is complex and depends on both modified residues and the state of methylation (Jambhekar et al., 2019). Additionally, histone phosphorylation, ubiquitylation, and ADP ribosylation are involved in the regulation of DNA damage and transcriptional activities (Liu C. et al., 2017; Alhamwe et al., 2018; Shanmugam et al., 2018).

### Chromatin Remodeling

Chromatin structure dynamics always correspond with the instructive gene expression pattern for cellular differentiation and lineage specification during development (Kishi and Gotoh, 2018; Quan et al., 2020). In addition to the covalent modification of DNA and histone, the remodeling of nucleosome is another important determinant of chromatin structure. Nucleosome formation, which is crucial for the compaction of genomic DNA into chromatin, has intrinsic dynamic properties regulated by chromatin remodeling complexes to ensure genomic DNA functions in chromatin (Zhou C. Y. et al., 2016; Kobayashi and Kurumizaka, 2019). Increasingly, reports of copious chromatin remodeling complexes and their essential regulatory potentials related to transcription activities and gene expression during development and disease processes have emerged (Bhattacharjee et al., 2016; Kim and Kaang, 2017; Stachowiak et al., 2020). Adenosine triphosphate (ATP)-dependent chromatin remodeling complexes are particularly known to utilize the energy derived from ATP hydrolysis to change nucleosome structure and consequently regulate DNA accessibility to TFs (Hota and Bruneau, 2016). As one representative of ATP-dependent complexes, BRM/BRG1-associated factor (BAF) complexes, also known as SWI/SNF (switch/sucrose non-fermentable) complexes, have various roles in gene activation and repression during mammalian development and in the development of disease (Clapier et al., 2017; Alfert et al., 2019; Hota et al., 2019). BAF complexes consist of over 15 different subunits with varied roles at different stages of mammalian development, including embryogenesis, neural development, cardiovascular development, skeletal muscle development, immune cell development, etc. (Hota and Bruneau, 2016; Nguyen et al., 2016; Sokpor et al., 2017; Sun et al., 2018). Homologous to the BAF complex, the RSC (remodel structure of chromatin) remodeling complex is an abundant and fundamental nuclear protein complex with important roles in transcriptional and other cellular processes, including initiation and elongation of transcription as well as replication, segregation, and chromosome repair (Klein-Brill et al., 2019; Ye et al., 2019). The RSC complex can partially disrupt histone–DNA interaction by stable binding to nucleosomes or enhancer elements and can also disassemble or slide nucleosome through a DNA-sequence-dependent system, that is required for nucleosome-free region formation by removing nucleosome from upstream of transcription start sites (TSSs; Spain et al., 2014; Lorch and Kornberg, 2017; Kubik et al., 2018). Besides, nucleosome remodeling and deacetylation (NuRD) complex (highly conserved in mammals), initially defined as a transcriptional repressor, has been reported to link histone modifications to nucleosome remodeling and interaction with numerous TFs (Feng and Zhang, 2003; Liang et al., 2017). Genome-wide data revealed the presence of the NuRD complex at all active enhancers and promoters in diverse cells, and also that NuRD could deposit histone modifications at enhancers and promoters of active genes and thereby trigger their repression (Miller et al., 2016; Yang et al., 2016a; Bornelöv et al., 2018).

### Non-coding RNA Regulation

In addition to these classic epigenetic processes (DNA methylation, histone modification, and chromatin remodeling), ncRNAs also play important regulatory roles in gene expression and chromatin modification impacting livestock production and health (Do et al., 2017a; Benmoussa et al., 2020). ncRNAs are a class of RNA species that mediate their functions as RNA (are not translated into proteins) and generally regulate gene expression at the transcriptional and posttranscriptional levels. ncRNAs with epigenetic-related functions interfere with transcription, mRNA stability, or translation and include small interfering RNA (siRNA), piwi-interacting RNA (piRNA), microRNA (miRNA), and long non-coding RNA (lncRNA; Kaikkonen et al., 2011; Wei et al., 2017). For example, some miRNAs regulated by *DNMT1* are involved in the regulation of mammary gland development and lactation in dairy cattle (Do et al., 2017b; Melnik and Schmitz, 2017a). The binding of miRNA to a specific target, resulting in degradation or blockage of mRNA transcription, may induce a feedback modification related to DNA methylation (Lamouille et al., 2014). Besides, ncRNAs have been found to be involved in the regulation of epigenetic alterations in both DNA and histones (Sabin et al., 2013). Furthermore, it is speculated that some transcripts initiating from gene body CpG islands are regulated by ncRNAs whose presence or absence affects the expression of the associated protein-coding genes or nearby genes (Mercer et al., 2009). For example, AIR is a ncRNA that initiates at a CpG island within intron 2 of *IGF2R* and is essential for silencing of the paternal allele (Sleutels et al., 2002). Similarly, analysis of a CpG island in intron 10 of the imprinted *KCNQ1* gene identified it as the origin of a non-coding transcript (KCNQ1OT1) that is required for imprinting of several genes within this domain (Mancini-DiNardo et al., 2006). ncRNA regulation impacts on livestock productivity will not be discussed in this review as it has been adequately covered in the literature and in recent reviews (Do and Ibeagha-Awemu, 2017; Wara et al., 2019; Kosinska-Selbi et al., 2020).

## Evidence of the Impacts of Epigenetic Processes on Livestock Production and Health

### Epigenetic Impacts on Livestock Reproduction, Growth, and Development

**FIGURE 1:**
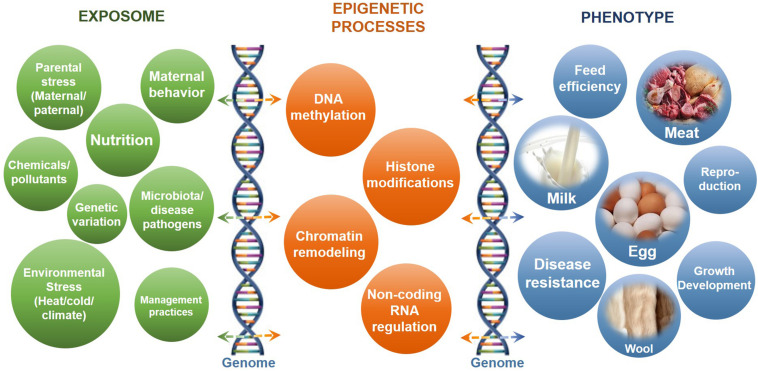
The impacts of epigenetic regulatory processes on livestock production in response to different impact factors or the exposome. Shown are the common impact factors or exposome **(left)** that interact with epigenetic processes **(center)** and the genome (vertical DNA helix structures) to influence phenotypic outcome **(right)**.

Dynamic epigenetic modifications are essential for normal growth and development through involvement in numerous biological processes, especially in response to environmental stimulus (Del Corvo et al., 2020; Thompson et al., 2020). The identification of epigenomic patterns in different tissues helps in the further understanding of epigenetic regulatory roles in livestock development and health. The impacts of epigenetic regulatory processes on livestock production in response to different impact factors or the exposome and the phenotypic outcomes are summarized in Figure 1. The important regulatory roles and impacts of epigenetic processes on placental and embryo development of livestock have been discussed in detail in many excellent reviews (Das et al., 2017; Franco, 2017; Hwang et al., 2017).

The genomic DNA methylation profiles of several tissues in multiple livestock species including cattle, chicken, swine, goat, sheep, etc. have been characterized (Korkmaz and Kerr, 2017; Lee K. H. et al., 2017; Zhang Y. et al., 2017; McKay et al., 2018; Sevane et al., 2019; Liu et al., 2020; Wang et al., 2020b). DNA methylation pattern screening in embryo at different embryonic stages of development, especially at the early stage when two major epigenetic reprogramming occur, indicated important regulatory roles of DNA methylation in embryo viability and fetus development relating to various metabolic and differentiation processes (Ispada et al., 2018; Salilew-Wondim et al., 2018; Duan et al., 2019; Cao P. et al., 2020; Ivanova et al., 2020). DNA methylation changes were found to partly explain the poor performance of offspring caused by maternal stressors, such as heat stress, metabolic disorder, and negative energy balance (Desmet et al., 2016; Akbarinejad et al., 2017). Differentially methylated cytosines (DMCs) in the liver from calves under maternal heat stress or cooling treatment (fans and water soakers) were found in genes involved in immune function, cell cycle, development, and enzyme activity, while DMCs in mammary gland tissues were enriched in biological functions, including protein binding, phosphorylation, enzyme and cell activation, and cell signaling (Skibiel et al., 2018). It was observed recently that sperm DNA methylome was characterized by generally low methylation levels compared with somatic tissue DNA methylome (Perrier et al., 2018; Zhou et al., 2018). Moreover, dysregulation of DNA methylation in sperm (Kropp et al., 2017; Perrier et al., 2018; Fang et al., 2019b) and histone modifications, including histone acetylation and methylation (Kutchy et al., 2017, 2018), were found to impact male fertility and related traits. For instance, sperm whole-genome bisulfite sequence (WGBS) data from high- and low-fertile bulls indicated high methylation differences (1,765 DMCs) and 10 candidate genes for the prediction of bull fertility (Gross et al., 2020). Another sperm WGBS dataset from 28 bulls further classified the sperm methylome into conversed, variable, and highly variable methylated regions (Liu S. et al., 2019). The highly variable methylated regions associated significantly with reproduction traits and were enriched for glycosyltransferase genes that are crucial for spermatogenesis and fertilization, while the variable methylated regions were co-localized with genes with functions in sperm motility (Liu S. et al., 2019). In addition, methylation analysis of high and low motile bull sperm populations found methylation variations in genes involved in chromatin remodeling and repetitive element activities in pericentric regions, which is indicative of crucial epigenetic regulatory functions in sperm functionality and fertility (Capra et al., 2019). Methylation alteration was also predicted as one potential epigenetic regulatory mechanism underlying sperm fertility differences caused by age and other stressors such as heat or oxidative stress (Lambert et al., 2018; Rahman et al., 2018; Wyck et al., 2018; Takeda et al., 2019). Moreover, epigenomic profiling of somatic tissues such as the liver, brain, and mammary gland tissues revealed that epigenetic modifications impact bovine development, health, and productivity (Zhou Y. et al., 2016; Kweh et al., 2019; Wang et al., 2020c). For example, DNA methylation is involved in the regulation of *SIRT6* promoter activity during bovine adipocyte differentiation (Hong et al., 2018).

Analysis of epigenetic processes in different porcine tissues, including tooth, brain, small intestine, and longissimus dorsi muscle, indicated their significant regulatory roles during the growth and development of pigs (Su et al., 2016; Larsen et al., 2018). The DNA methylation patterns of porcine tooth germ from different developmental stages (embryonic day 50 and day 60) revealed 2,469 differentially methylated genes (DMGs), including 104 DMGs with potential key regulatory roles in porcine tooth development (Su et al., 2016). Using the whole-genome DNA methylation approach to profile DNA methylation of porcine longissimus dorsi muscles from heat-stressed and non-stressed pigs, Hao and colleagues identified 57,147 differentially methylated regions (DMRs) and corresponding DMGs (*n* = 1,422) with functions in energy and lipid metabolism, cellular defense, and stress responses, indicating the roles of DNA methylation in heat stress processes (Hao et al., 2016). The expressions of *DNMT1*, *DNMT3A*, and *DNMT3B* were found to decrease in brain tissues during the middle stage of gestation, indicating the potential of DNA methylation to regulate brain development of piglets (Larsen et al., 2018). Daily oral boluses of broad-spectrum antibiotics after preterm birth decreased bacterial density, diversity, and fermentation and altered the DNA methylation profile in the small intestine, which is indicative of the influence of epigenetic process on bacterial colonization of preterm neonate’s intestine (Pan et al., 2018). Furthermore, transcriptional N6-methyladenosine (m^6^A) profiling in porcine liver at different ages (0 day, 21 days, and 2 years) demonstrated that m^6^A modified about 33% of transcribed genes with roles in the regulation of growth and development and metabolic and protein catabolic processes implying that m^6^A methylation may be vital for the regulation of nutrient metabolism in porcine liver (He et al., 2017). Moreover, abundant m^6^A modifications were identified in granulosa cells, which are crucial for follicle development with potential associations with steroidogenesis and folliculogenesis in pigs (Cao Z. et al., 2020).

DNA methylation and histone modification patterns have been characterized in various chicken strains in recent years, indicating the potential roles of epigenetic processes in the development and evolution of chicken (Li et al., 2015; Sarah-Anne et al., 2017). Epigenetic analysis of various tissues including the brain, retina, cornea, liver, and muscle strongly revealed the involvement of DNA methylation in the growth and development of chicken (Liu et al., 2016, Liu Z. et al., 2019; Lee I. et al., 2017). For example, DNA methylation profiles of broilers and layers at different embryonic stages revealed lower methylation levels in broilers, and enriched gene ontology terms related to muscle development by corresponding genes suggest a potential contribution of DNA methylation to embryonic muscle development (Liu Z. et al., 2019). *ACC* and *MTTP* showed abundant expression and were negatively correlated with lower DNA methylation at their promoter regions in the liver of chickens with fatty liver syndrome, linking DNA methylation to fat metabolism (Liu et al., 2016). The global DNA methylation profiling of strongly and weakly inbred chickens identified various DMRs and DMGs enriched in reproduction pathways, indicating the regulatory roles of DNA methylation in the repressed development of the reproduction system of inbred chickens (Han et al., 2020). Besides, chicken erythrocyte epigenome analysis identified more than 100 highly transcribed genes located in dynamical highly acetylated, salt-soluble chromatin domains, which were associated with H3K4me3 and H3K27ac, and also produced distinct antisense transcripts (Jahan et al., 2016). Results of comparing histone H1 subtypes between five avian species (chicken, gray partridge, quail, duck, and pheasant) indicated the potential involvement of histone modification in chromatin structure and function in the development of poultry (Kowalski and Pałyga, 2017).

In addition to the main livestock species discussed above, some epigenetics-related data have been reported in other livestock species. For instance, DNA methylation was predicted to be an age-dependent process in domestic horses (Andraszek et al., 2016). Different from the generally methylated mammalian genomes, honeybees have unique methylation patterns that concentrate in gene bodies and are associated with gene expression (Wedd and Maleszka, 2016; Harris et al., 2019). DNA methylation is involved in the learning and memory processes of honeybees, crucial traits for honey production (Li et al., 2017). Besides, epigenetic modifications, including DNA methylation, histone methylation, and phosphorylation, represent new possible mechanisms of sex and caste determination process in honeybees (Cardoso-Júnior et al., 2017; Yagound et al., 2019). Moreover, WGBS data showed differential methylation levels between honeybee queen larvae and worker larvae as well as 38 DMGs with functions in specific organ differentiation, morphology, reproduction, and vision differentiation during caste determination (Wang et al., 2020a). Furthermore, the identification of allele-specific DNA methylation patterns in honeybees provided a relatively reliable theory of genomic imprinting underlying parent-of-organ effects caused by reciprocal crosses (Remnant et al., 2016).

### Epigenetic Regulation in Response to Nutritional Stimulus

Nutrition represents the principal environmental determinant of an individual’s growth and development. In order to enhance livestock health and welfare, reduce production cost, and adapt to global warming, efforts have been concentrated on adjusting nutritional supplements to livestock animals and their related impacts (McGuffey, 2017; Bobeck, 2020). Growing evidence supports the notion that permanent alterations in the epigenome of germline cells or embryos could be transferred to offspring, referred to as intergeneration or transgenerational epigenetic inheritance (Heard and Martienssen, 2014; Miska and Ferguson-Smith, 2016). It is well accepted that nutrition-induced epigenetic alterations can be heritable; however, the underlying mechanism is still controversial. On one side, epigenetic changes caused by consistent nutritional stimulus were identified in somatic tissues, indicating an undefined indirect mechanism of inheritance (Xue et al., 2016). On the other side, continuous stimulus as a result of the effects of nutritional factors on health and diseases of livestock could be transmitted between generations through altered epigenetic state of germline cells (Ideraabdullah and Zeisel, 2018). For example, the excessively high or excessive lack of nutrition (hyper-/hyponutrition, respectively) or nutrition component deficiencies could lead to epigenetic alterations (DNA methylation, histone modifications, and ncRNAs) in germ cells and transmission to subsequent generations (Guo T. et al., 2020). A number of studies, both *in vivo* and *in vitro*, showed that nutritional stimulus, including methionine, choline, and energy restriction, could induce epigenetic modifications causing the alteration of gene expression (Murdoch et al., 2016; Chavatte-Palmer et al., 2018; Elolimy et al., 2019). Data on the epigenetic modifications in response to nutritional stimulus in livestock are summarized in Table 1.

**TABLE 1:** Epigenetic alterations in response to nutritional stimulus in livestock.

Parameter	Breed	Organ	Epigenetic alteration	*Potential epigenetic marker genes	References
**Cattle**					
Methionine supplementation throughout the peripartal period	Holstein cows	Liver	Global DNA methylation was lower, promoter methylation of *PPARA* was higher	*PPARA*	Osorio et al., 2016
Grass-fed and grain-fed	Angus cattle	Rumen tissue	217 DMRs	*ADAMTS3*, *ENPP3*	Li Y. et al., 2019
High- vs low-concentrate corn straw (HCS/LCS) and low-concentrate mixed forage diet	Chinese Holstein cows	Mammary tissue	H3 acetylation reduced in HCS. Methylation of *STAT5A* reduced in HCS. Methylation of *SCD* increased in HCS	*STAT5A*	Dong et al., 2014
High- vs low-concentrate diet	Holstein cows	Liver	Chromatin loosening at the promoter region	*TLR4*, *LBP*, *HP*, *SAA3*	Chang et al., 2015
Methyl donor supplementation of pregnant animals	Holstein cows and calves	Blood	More than 2,000 DMG between offspring	*CEND1*, *VSIG2*, *B3GNT8*, etc.	Bach et al., 2017
85 vs 140% feed restriction	Angus–Simmental crossbred cows	*Longissimus dorsi* and *semitendinosus* muscles	One DMR (contains 23 to 24 CpGs) in *IGF2*	*IGF2*	Paradis et al., 2017
Butyrate treatment	Holstein cows	Rumen primary epithelial cells from 2-week-old bull calves	Increased genome coverage (%) of CTCF, H3K27me3, and H3K4me3 and decreased coverage of H3K27ac, H3K4me1, and ATAC	Weak enhancers and flanking active transcriptional start sites	Fang et al., 2019a
**Swine**					
Restricted diet	Pig	Endometrium and embryos	Altered DNA methylation of selected genes	*ACP5*, *RGS12*, *EDNRB*, *TLR3*, *ADIPOR2*, *DNMT1*	Zglejc-Waszak et al., 2019
Vitamin C supplementation	Pig	Oocyte	Methylation erasure on 5mC, m^6^A, and H3K27me3, but establishment of H3K4me3 and H3K36me3	*TET2*, *DNMT3B*, *HIF-1*α, *HIF-2*α, *TET3*, *METTL14*, *KDM5b*, and *EED*	Yu et al., 2018
Omega-3 fatty acid supplementation	Piglet	Leukocyte	One hypomethylated DMR in chromosome 4, two hypermethylated DMRs in chromosomes 4 and 12	*RUNX1T1*	Boddicker et al., 2016
Maternal bisphenol A and methyl donor supplementation	Piglets from Landrace × Yorkshire sows	Piglet intestinal samples	Altered DNA methylation level of *PEPT1* in jejunum of offspring	*PEPT1*, *DNMT3a*, *LCT*, *DNMT1*, *MTHFR*	Liu H. et al., 2017
Maternal methyl donor dietary supplementation	Piglets from White × Landrace sows	Liver samples from piglets	Increased methylation at the promoter of *IGF-1* in the methyl donor group	*IGF−1*	Jin C. et al., 2018
Maternal betaine supplementation	Piglets from Landrace × Yorkshire sows	Liver samples from piglets	DNA hypermethylation and enriched H3K27me3 on the promoter region	*GALK1*	Cai et al., 2017
**Poultry**					
Maternal genistein supplementation	Laying broiler breeder hens	Liver from pullets	Induced H3K36me3 and H4K12ac at the *PPARD* promoter	*PPARD*	Lv et al., 2019
Prenatal betaine supplementation	Juvenile chicken	Liver from juvenile chicken	Increased CpG methylation at promoter regions	*LXR* and *CYP27A1*	Hu et al., 2017b
Maternal betaine supplementation	Rugao Yellow chicken breeder hens	Hypothalamus of F1 cockerels	Modification of promoter CpG methylation	*DNMT1*, *BHMT SREBP-1*, *SREBP-2*, and *APO-A1*	Idriss et al., 2018
Maternal betaine supplementation	Rugao yellow-feathered laying hens	Blood from pullets	Hypomethylated promoter regions of steroidogenic genes	*AHCYL*, *GNMT1*, and *BHMT*	Abobaker et al., 2019
Methionine and betaine supplementation	Geese	Liver	Altered DNA methylation of *LOC106032502*	*LOC106032502*	Yang et al., 2018

*Potential epigenetic marker genes: These are genes harboring differentially methylated CpG sites or regions; vs, versus; DMR, differential methylated region; DMG, differential methylated gene; DMC, differential methylated CpG; HCS, high-concentrate corn straw; LCS, low-concentrate corn straw; CTCF, CCCTC-binding factor; ATAC, Ada-Two-A-Containing complex; H3K27me3, the trimethylation at the 27th lysine residue of the histone H3 protein; H3K4me3, the trimethylation at the fourth lysine residue of the histone H3 protein; H3K27ac, the acetylation at the 27th lysine residue of the histone H3 protein; H3K4me1, the monomethylation at the fourth lysine residue of the histone H3 protein; H3K36me3, the trimethylation at the 36th lysine residue of the histone H3 protein; H4K12ac, the acetylation at the 12th lysine residue of the histone H4 protein.

DNA methylation modifications in response to nutritional stimulus or environmental changes may cause alteration in production performance or disease susceptibility (Jang and Serra, 2014; Block and El-Osta, 2017; Maugeri and Barchitta, 2020). The interaction between changes in feed composition and epigenetic regulatory mechanisms has been reported. Liver tissues of methionine (Met)-supplemented Holsteins were found to have lower general DNA methylation levels compared with that of Holsteins without Met supplementation (Osorio et al., 2016). In the same study, the overall gene expression levels of *PPAR*α and its target genes were upregulated in Met-supplemented Holsteins, which was related to improved metabolism and immune functions (Osorio et al., 2016). Additionally, differential expression of *ADAMTS3* and *ENPP3* genes (have roles related to the biosynthesis and regulation of glycosyltransferase activity, respectively) between grass-fed and grain-fed Angus cattle were associated with the methylation abundance of corresponding DMRs (Li Y. et al., 2019). Meanwhile, DNA methylation was involved in the regulation of altered gene expression in response to high-concentrate diets resulting in the downregulation of immune-related genes (*TLR-4*, *LBP*, *HP*, and *SAA3*) in mammary and liver tissues of cows (Dong et al., 2014; Chang et al., 2015; Xu et al., 2018). In addition to DNA methylation, other epigenetic modifications have been reported to respond to nutritional stimulus. Histone H3 acetylation was significantly reduced in mammary gland tissue and also correlated negatively with lipopolysaccharide (LPS) concentrations in the mammary arterial blood of Chinese Holstein cows fed a high-concentrate corn straw diet (Dong et al., 2014). Linseed oil supplementation of Holstein cows in mid lactation, which resulted in 30% reduction of milk fat yield, significantly repressed the expression of histone acetylases (*HDAC2*, *HDAC3*, *SIRT2*, and *KAT2A*) and histone methyltransferase (*EHMT2*), suggesting potential epigenetic regulation of milk fatty acid synthesis (Li and Ibeagha-Awemu, 2017). Additionally, chromatin loosening was found to contribute to the upregulation of some immune-related genes in the liver of dairy cows in response to a high-concentrate diet (Chang et al., 2015). Furthermore, butyrate treatment of rumen epithelial cells revealed increased genome coverage (percentage) of CTCF, H3K27me3, and H3K4me3, but decreased coverage of H3K27ac, H3K4me1, and ATAC (Fang et al., 2019a). In the same study, 15 distinct chromatin states were defined according to the combination of identified epigenomic markers in genomic regions, which revealed that weak enhancers flanking active transcriptional start sites could be possible mechanisms underlying gene expression regulation by epigenomic markers (Fang et al., 2019a).

Nutritional supplementation during pregnancy caused epigenetic alterations in the fetus with long-term influences on the development and productivity of the offspring. Embryos (at 6.5 days) of dairy cows showed lower DNA methylation level in response to Met supplementation during the preimplantation period, which probably enhanced its survival capacity (Acosta et al., 2016). The supplementation of methyl donors to Holstein dams during pregnancy significantly altered the methylome of their offspring, and the DMCs affected the expression of genes involved in various biological processes, such as immune function, regulation of cell growth, and kinase activity (Bach et al., 2017). Maternal methyl donor supplementation was also found to alter the hepatic metabolism program of calves by maintaining Met homeostasis, DNA methylation, energy metabolism, etc., which potentially contributed to better nutrient utilization efficiency of calves and promoted their growth and development performance (Alharthi et al., 2019). Moreover, energy restriction significantly impacted the DNA methylation level of a DMR in *IGF2* in fetal longissimus dorsi of beef cattle, where *IGF2* expression was negatively associated with fetus weight in Angus–Simmental crossbred cows (Paradis et al., 2017). Furthermore, offsprings’ weight was affected by their mothers’ high-fat diet (offspring were obese), and individual differences of obesity were potentially regulated by epigenetic modifications (Keleher et al., 2018; Glendining and Jasoni, 2019). Also, 5hmC and 5mC were found to be negatively and positively correlated with body weight in offspring, respectively, and altered CpG methylation in the proopiomelanocortin (*POMC*) promoter region induced histone modification through binding of MBD1 to 5mC, which reduced *POMC* expression (Marco et al., 2016). Furthermore, it was reported that changes in diet during pregnancy can also influence the reproduction ability of female offspring, which may be regulated by epigenetic modifications (Noya et al., 2019; Shah and Chauhan, 2019).

In pigs, dietary changes, such as feed restriction and vitamin C supplemental feeding, were reported to induce modifications of DNA methylation during development of porcine germline cell and embryo (Yu et al., 2018; Zglejc-Waszak et al., 2019). Prenatal and postnatal dietary omega-3 fatty acid supplementation resulted in altered global DNA methylation patterns and probable implication in the growth and inflammatory processes of piglets (Boddicker et al., 2016). In addition, supplementation of methyl donors during gestation could improve intestinal digestion and absorption and the growth rate of offspring piglets, and these attributes were associated with DNA methylation modifications in specific genes and their corresponding regulated expression (Liu H. et al., 2017; Jin C. et al., 2018). Furthermore, repressed expression of *GALK1* gene by DNA hypermethylation and histone trimethylation in the liver was associated with low serum concentration of galactose in neonatal pigs in response to betaine-supplemental feeding of sows (Cai et al., 2017).

Epigenetic modifications have been reported to regulate the impacts of parental feed additive supplementation on offspring pullets. For example, histones H3K36me3 and H4K12ac in the promoter region of *PPAR*δ gene were involved in the regulation of altered lipid metabolism and growth performance following maternal genistein supplementation (Lv et al., 2019). Betaine is a frequently used supplement in the chicken industry, and its impact on intercellular metabolism is probably influenced by DNA methylation (Hu et al., 2017a). DNA methylation alteration was found to regulate gene expression related to cholesterol and corticosteroid synthesis of offspring pullets in response to maternal betaine supplementation (Hu et al., 2017b; Idriss et al., 2018; Abobaker et al., 2019). Additionally, DNA methylation was associated with the regulation of transcriptional regulatory network in response to dietary methionine and betaine supplementation in goose (Yang et al., 2018).

### Epigenetic Regulation of Livestock Products

#### Milk

Milk production is the most important economic trait of the bovine dairy industry, which is affected by multitudinous factors including genetics, nutrition, health, farm management, and environmental conditions (Pragna et al., 2017; Waterman et al., 2017; Sørensen et al., 2018). As summarized in Table 2, epigenetic modifications have been identified as important regulatory mechanisms of milk production in dairy cows and other livestock species (Singh et al., 2010, 2011; Ibeagha-Awemu and Zhao, 2015). Significant differences of global DNA methylation levels in blood were reported between lactating dairy cows with high and low milk yield, indicating the association between milk yield and DNA methylation (Dechow and Liu, 2018; Wang H. et al., 2019). Particularly, abnormal DNA methylation around the STAT5-binding enhancer in the αS1-casein promoter negatively regulated αS1-casein synthesis in milk during lactation, which could be affected by foreign stimulus, such as mastitis and daily milking times (Platenburg et al., 1996; Vanselow et al., 2006; Nguyen et al., 2014). Additionally, the DNA methylation of *EEF1D*, a gene strongly related to milk production, regulates its spatial expression (Liu X. et al., 2017). Meanwhile, differential DNA methylation levels of milk-related genes (e.g., *PPAR*α, *RXR*α, and *NPY*) in the mammary glands of dairy goats at dry and lactation periods indicated important regulatory roles of DNA methylation in goat lactation (Zhang X. et al., 2017). Moreover, inhibition of miR-145 expression impaired fatty acid synthesis in goat milk by increased methylation levels of some lipid-related genes (*FASN*, *SCD1*, *PPARG*, and *SREBF1*) (Wang et al., 2017b). In addition, higher promoter DNA methylation of *ACACA* and *SCD* downregulated their expression and were associated with decreased milk fat of dairy goats in response to a high grain diet (Tian et al., 2017).

**TABLE 2:** Alteration of epigenetic markers in relation to livestock production traits.

Parameter	Breed	Organ	Epigenetic alteration	Production trait	Model and references
**Milk**					
High and low milk yield	Holstein cows	Peripheral blood mononuclear cells	72 DMRs between high and low milk yield, 252 DMRs across herd environments	Milk yield	Dechow and Liu, 2018
High and low milk yield	Holstein cows	Jugular venous blood	DNA methylation rates in the lower-yield cows were significantly higher than those in the higher-yield animals	Milk and protein yield	Wang L. et al., 2019
Experimentally induced mastitis by *E. coli* and *S. aureus*	Holstein cows	Liver and mammary gland tissues	Remethylation of upstream promoter of *CSN1S1* gene in response to *E. coli* infection	α*S1*-casein synthesis	Vanselow et al., 2006
Once/twice daily milking	Holstein cows	Liver and mammary gland tissue	Altered methylation of four CpG within the distal upstream regulatory region of the *CSN1S1* gene	Milk production (yield and milking frequency)	Nguyen et al., 2014
Different lactation stages	Holstein cows	Blood	DNA methylation of *EEF1D* was lower in the dry period than the early stage of lactation	Milk production	Liu X. et al., 2017
Different lactation stages	Xinong Saanen goats	Mammary gland tissue	Methylation levels of 95 and 54 genes in the lactation period were up- or downregulated, respectively, relative to the dry period	Milk production	Zhang X. et al., 2017
Different lactation stages	Xinong Saanen goats	Mammary gland tissue	Inhibition of miR−145 increased methylation levels of *FASN*, *SCD1*, *PPARG*, and *SREBF1*	Milk fat synthesis	Wang et al., 2017b
High- and low-concentrate diet feeding	Guanzhong goats	Mammary gland tissue	Increased DNA methylation level in the promoter regions of the *ACACA* and *SCD* genes	Milk fat production and composition	Tian et al., 2017
**Meat**					
Two sheep breeds differing in meat production ability	Small-tailed Han and Dorper × small-tailed Han crossbred sheep	*Longissimus dorsi* muscles	808 DMRs and global loss of DNA methylation in the DMRs in the crossbred sheep, 12 potential DMGs	Meat production	Cao et al., 2017
Two cattle breeds exhibiting different meat production ability	Japanese black and Chinese Red Steppes cattle	*Longissimus dorsi* muscles	23,150 DMRs identified, 331 DMRs correlated negatively with expression of DE genes, 21 DMRs located in promoter regions	Muscle development and related meat quality traits	Fang et al., 2017
Divergent beef tenderness	Angus beef	*Longissimus dorsi* muscles	DNA methylation profiles related to beef tenderness, and 7215 DMRs between tender and tough beef	Beef tenderness	Zhao et al., 2020
Three cattle breeds differing in meat production abilities	Simmental, Yunling, and Wenshan cattle	*Longissimus dorsi* muscles	18 DM and DE genes between Simmental and Wenshan cattle, 14 DM and DE genes between Simmental and Yunling cattle, 28 DM genes between Wenshan and Yunling cattle	Meat quality	Chen Z. et al., 2019
Three growth stages	Polled yak	*Longissimus dorsi* muscles	1,344, 822, and 420 genes with DM CCGG sites and 2,282, 3,056, and 537 genes with DM CCWGG sites between 6-month-old vs 90-day-old, 6-month-old vs 3-year-old, and 3-year-old vs 90-day-old fetuses, respectively	Muscle development and growth	Ma et al., 2019
Feed restriction	Angus–Simmental crossbred cows	*Longissimus dorsi* and *semitendinosus* muscle	One DMR in *IGF2*	Muscle function	Paradis et al., 2017
Fetal and adult cattle	Qinchuan cattle	*Longissimus dorsi* muscles	Three DMCs in the core promoter region of *SIX1*, histone H4 and E2F2 bind to *SLX1*	Muscle development	Wei et al., 2018
Obese, lean, and miniature pig breeds	Tongcheng, Landrace, and Wuzhishan pigs	Blood leukocytes	2,807, 2,969, and 5,547 DMGs in the Tongcheng vs Landrace, Tongcheng vs Wuzhishan, and Landrace vs Wuzhishan comparisons, respectively	Fat-related phenotype variance	Yang et al., 2016b
Obese and lean type pig breeds	Landrace pigs and Rongchang pigs	Backfat	483 DMRs in the promoter regions	Fat deposition and fatty acid composition	Zhang S. et al., 2016
Castrated and non-castrated pigs	Male Huainan pigs	Liver and adipose tissues	*GHR* methylation rate in the liver of castrated and non-castrated pigs were 93.33% and 0, respectively	Castration-induced fat deposition	Wang et al., 2017e
Pig breeds differing in metabolic characters	Duroc and Pietrain	*Longissimus dorsi* muscles	More than 2,000 DMCs	Muscle metabolism	Ponsuksili et al., 2019
Three pig breeds differing in fatness traits	Polish Large White, Duroc and Pietrain	Subcutaneous fat, visceral fat, and *longissimus dorsi* muscle	H3K9ac and H3K4me3 correlated to the expression level of selected genes	Adipose tissue accumulation	Kociucka et al., 2017
Highest and lowest pH among littermates	Duroc	*Longissimus dorsi* muscle	3,468 DMRs, including 44 and 21 protein-coding genes with hyper- and hypomethylation regions in their gene bodies	Postmortem energy metabolism and pH	Park et al., 2019
High and low boar taint	Pigs	Testis	32 DE genes with DMCs	Boar taint	Wang and Kadarmideen, 2019a,c
Different growth stages	Gushi hens	Breast muscle	2,714 DMRs and 378 DMGs	Intramuscular fat deposition and water-holding capacity	Zhang M. et al., 2017
Different feed conditions and breeds	Daninghe and Qingjiaoma chickens	Breast muscle	46 CpG sites and 3 CpG islands in *UCP3*, different methylation levels of *UCP* and *FATP1* between groups	Breast muscle (intramuscular fat content)	Gao et al., 2015, 2017
**Egg**					
Before and after reproductive maturation	Hy-Line Brown commercial female chickens	Ovaries	Increased methylation of two CpG sites in ERα; increased H3K27ac and decreased H3K36me3 related to increased ERα mRNA transcript	Reproductively mature, egg production	Guo M. et al., 2020
Betaine supplementation	Laying hens	Liver	Hypomethylation of promoter in *GR*	Egg production	Omer et al., 2018, 2020
**Wool**					
Different generations of cashmere goats	Cashmere goats	Skin	336 hyper- and 753 hypomethylated 5mC, corresponding to 214 hyper- and 560 hypomethylated genes	Cashmere traits	Dai et al., 2019
Anagen and telogen stages	Cashmere goats	Skin	1,311 DMRs corresponding to 493 DMGs (269 hyper- and 224 hypomethylated DMGs)	Hair cycling and cashmere growth	Li et al., 2018
Coarse type and fine type cashmere	Cashmere goats	Skin	9,085 DM N6-methyladenosine sites	Cashmere fiber growth	Wang et al., 2020f
Cashmere goats and other goat species	Cashmere goats	Skin	Altered methylation degree of *HOXC8* exon 1	Cashmere fiber growth	Bai et al., 2017
Anagen and telogen stages	Cashmere goats	Skin	Altered promoter methylation of *HOTAIR* gene	Cashmere fiber growth	Jiao et al., 2019

*DM, differentially methylated; DE, differentially expressed; DMR, differential methylated region; DMG, differential methylated gene; DMC, differential methylated CpG; H3K9ac, histone H3 lysine 9 acetylation; H3K4me3, trimethylation at the fourth lysine residue of histone H3 protein; H3K27ac, acetylation at the 27th lysine residue of histone H3 protein; H3K36me3, trimethylation at the 36th lysine residue of histone H3 protein.*

Interesting reports of how DNA methylation interacts with miRNA expression and function to regulate milk production have emerged. MiR-152 and miR-29s and their respective target genes *DNMT1*, and *DNMT3A*, and *DNMT3B* are inversely expressed during lactation (Bian et al., 2015; Melnik et al., 2016). For example, miR-148a and miR-152 as well as miRNA-29s impact bovine mammary gland epithelial cell activities and milk synthesis by reducing the mRNA expression levels of *DNMT1* as well as *DNMT3A* and *DNMT3B*, respectively (Wang et al., 2014; Melnik and Schmitz, 2017a; Liang et al., 2018). Specifically, over- or forced expression of miR-152 resulted in marked reduction of DNMT1 expression (both mRNA and protein), decrease in global DNA methylation levels, increase in the expression of two lactation-related genes (*AKT* and *PPAR*γ), and enhanced viability and multiplication capacity of mammary epithelial cells (Wang et al., 2014). These effects were reversed by inhibition of miR-152 expression (Wang et al., 2014). Similarly, inhibition of miR-29s triggered global DNA hypermethylation and increased methylation levels of the promoters of some important lactation-related genes, such as *CSN1S1*, *ElF5*, *SREBP1*, *PPAR*γ, and *GLUT1*, and consequently decreased the secretion of triglycerides, lactose, and lactoprotein by cow mammary gland epithelial cells (Bian et al., 2015). MiRNAs targeting *DNMTs* were also found to decrease the methylation of core CpG islands at the promoter regions of genes (such as *FTO*, *INS*, *IGF1*, *CAV1*, etc.) involved in the activation or regulation of various genes with roles in metabolism and milk synthesis (Melnik and Schmitz, 2017b). Conversely, induced methylation at the 5′ terminal of miR-183 inhibited its expression, consequently affecting milk lipid metabolism of dairy cows (Jiao et al., 2020). Furthermore, milk exosomes, regarded as epigenetic regulators that transfer specific regulatory molecules to consumers, regulate the expression of *DNMTs* and affect human health, especially milk allergy (Melnik et al., 2016; Paparo et al., 2016; Melnik and Schmitz, 2017b).

#### Meat

DNA methylation is one of the most studied epigenetic mechanisms involved in the regulation of gene expression related to muscle development (Table 2; Baik et al., 2014; Gotoh, 2015; Chen Z. et al., 2019). The genome-wide DNA methylation profiles of longissimus dorsi muscles from different breeds of sheep provided insight on the epigenetic regulatory mechanisms modulating the expression of genes involved in the regulation of muscle development, such as *DLK1*, *NR4A1*, *TGFB3*, *ACSL1*, *RYR1*, *ACOX2*, *PPARG2*, *NTN1*, and *MAPRE1* (Couldrey et al., 2014; Cao et al., 2017; Fan et al., 2020). Meanwhile, a number of DMRs on genes associated with important biological processes such as lipid translocation and lipid transport were identified in latissimus dorsi muscle from different breeds of beef cattle with diverse meat quality traits (Fang et al., 2017). For instance, DNA methylation profiling in relation to beef tenderness in Angus cattle revealed 7,215 DMRs between tender and tough beef (Zhao et al., 2020). The DMRs were significantly enriched in ATP binding cassette subfamily and myosin-related genes, including *ABCA1*, *ABCA7*, and *ABCG1*, with roles in beef tenderness and fatty acid metabolism (Zhao et al., 2020). Besides, demethylation of a DMR in the *SIRT4* promoter promoted its transcriptional activity through *CMYB* mediation or inhibited its transcriptional activity through *NRF1* mediation, thereby shedding light on the role of an epigenetic process in the transcriptional regulation of the expression of *SIRT4* during bovine adipocyte differentiation (Hong et al., 2019). In addition, important genes with DMRs, including *TMEM8C*, *IGF2*, *FASN*, *CACNA1S*, *FADS6*, and *MUSTN1*, were significantly associated with differences in muscle development and meat quality in several cattle (beef) breeds (Chen Z. et al., 2019; Ma et al., 2019). The methylation level of *IGF2*, which negatively correlated with its expression, was found to change more in longissimus dorsi muscle than in semitendinosus muscle in response to feed restriction (Paradis et al., 2017). Besides, differential expression of some important DNA methylation genes (*DNMT3A*, *DNMT3B*, and *DNMT1*) were significantly associated with meat and carcass quality traits such as carcass weight, flank thickness, and chuck short rib score in Wagyu × Limousin × Fuzhou yellow crossbred beef cattle (Guo et al., 2012; Liu et al., 2015). DNA methylation in the core promoter region of *SIX1* gene in muscle tissues was potentially regulated by histone H4 and *E2F2* and shown to impact muscle development in Qinchuan cattle (Wei et al., 2018). Expression and splicing quantitative trait loci mapping analyses for meat quality traits in longissimus dorsi muscle found that the expression of *PHF14*, an important epigenetic regulator of organ development, was influenced by multigenic effects (Leal-Gutiérrez et al., 2020). The PHF14 protein has many plant homeodomain fingers that are able to recognize specific epigenetic markers on histone tails and thus regulate gene expression, indicating its important roles in skeletal muscle growth and development.

Meat quality is also a trait of high interest in the pig industry. Genome-wide DNA methylation analysis identified numerous DMRs and DMGs between obese and lean pigs, revealing vital roles of DNA methylation in lipogenesis in pigs (Yang et al., 2016b). Specifically, the back fat of Landrace pigs (leaner) had higher global DNA methylation level than the fatty Rongchang pigs, indicating that some identified DMRs may affect lipid metabolism (Zhang S. et al., 2016). Altered methylation in lipid metabolism-related genes was also identified in diverse tissues from different pig breeds (Wang et al., 2017e; Ponsuksili et al., 2019). Additionally, histone modifications were found to affect adipose tissue accumulation by regulating corresponding gene expression (Kociucka et al., 2017). Moreover, DNA methylation pattern scanning revealed its potential involvement in other meat quality traits, such as pH, meat color, and carcasses’ traits (Te Pas et al., 2017; Park et al., 2019). Boar taint, an unpleasant odor that affects pork acceptability, was found to be regulated by epigenetic processes. Genome-wide methylation analysis of the testis of pigs with high, medium, and low boar taint associated DMCs and candidate genes with pig reproduction (e.g., *DICER1*, *PCK1*, *SS18*, and *TGFB3*) and boar taint (e.g., *CAPN10*, *FTO*, *HSD17B2*, *IGF2*, *SALL4*, *FASN*, *PEMT*, *CRYL1*, *DNMT3A*, and *EGFR*) thereby revealing important regulatory roles of DNA methylation in boar taint formation (Wang and Kadarmideen, 2019a,b).

In chickens, DNA methylation was reported as one of the regulatory mechanisms modulating crucial meat traits such as intramuscular fat deposition and skeletal muscle development (Zhang M. et al., 2017, Zhang et al., 2020). The whole-genome DNA methylation profiles of later laying-period hen and juvenile hens with differential intramuscular fat deposition and water-holding capacities identified 378 DMRs related to muscle development (Zhang M. et al., 2017). Further research indicated that DNA methylation affected the intramuscular fat deposition by regulating the expression of some key genes, such as *ABCA1*, *COL6A1*, and *GSTT1L* (Zhang M. et al., 2017, Zhang et al., 2020). Moreover, different breeds or feed condition significantly affected the methylation levels of *UCP3* and *FATP1* genes in chicken breast muscle, which enhanced the reliability of these genes as important candidate genes of intramuscular fat deposition in chicken meat (Gao et al., 2015, 2017). The alterations of epigenetic markers in relation to livestock products (milk, meat, egg, and wool) are summarized in Table 2.

#### Egg

Egg laying in poultry relies on the reproductive maturation of the ovaries, where epigenetic mechanisms play important regulatory roles (He et al., 2018). Epigenetic modifications in *ER*α were identified during ovarian development and maturation, whereby higher DNA methylation rates in specific CpG sites, higher histone H3K27ac, and lower H3K36me3 associated the abundance of *ER*α expression with important roles in egg laying (Guo M. et al., 2020). In addition, changes in DNA methylation were identified in response to betaine supplementation and were associated with improved egg laying performance in hens (Xing and Jiang, 2012). Supplemental betaine potentially caused hypomethylation of the promoter of *GR*, followed by enhanced expression of *GR* and *GR/ER*α interaction (contributed to increase *VTGII* expression in the liver), which partly supported improved egg production in betaine-supplemented laying hens (Omer et al., 2018). Furthermore, promoter region methylation could be the possible regulatory mechanism underlying altered expression of liver lipid synthesis and transport-related genes in response to betaine supplementation, which supported the synthesis and release of yolk precursor substances in the liver and consequently promoted egg laying performance (Omer et al., 2020).

#### Wool

Wool is an economic product of high regard with increasing value in the goat industry, but the limited yield of cashmere wool (or cashmere) was recently speculated to be potentially regulated by epigenetic modifications (Wang et al., 2017f, 2020d). DNA methylation and histone acetylation were found to actively contribute to the regulation of goat fetal fibroblast cells, which is critical for cashmere production (Wang et al., 2017f; Palazzese et al., 2018). Recently, DNA methylation was associated to the genetic stability of cashmere traits between generations of cashmere goats (Dai et al., 2019). Besides, genome-wide scanning revealed potential regulatory roles of DNA methylation and RNA m^6^A methylation in the growth and development of cashmere fibers in cashmere goats (Li et al., 2018; Wang et al., 2020f). Moreover, epigenetic modifications of some specific genes were reported to affect cashmere traits. For instance, methylation of *HOXC8* is involved in regulating the growth of cashmere fiber in cashmere goat (Bai et al., 2017). Promoter methylation of *HOTAIR* gene and related suppressed expression were found to regulate the reconstruction of secondary hair follicles in cashmere goat (Jiao et al., 2019). Furthermore, crucial regulatory roles for DNA methylation in wool fiber development and transformation of fur with special characters and production purpose, such as curly wool with beautiful white color or high-quality brush hair, have been observed (Qiang et al., 2018; Xiao et al., 2019).

### Epigenetic Regulation of Livestock Health

#### Epigenetic Regulation of Livestock Response to Environmental Stress

Environmental stressors including heat stress, pathogens, dietary changes, etc. are the greatest determinants of individual health and productivity. As summarized in Table 3, epigenetic alterations in response to environmental stressors have been reported in livestock animals. Heat stress has negative impacts on animal production and health, which may continue to be of great concern due to increasing global temperatures. The important roles of DNA and histone methylation on heat-shock proteins under heat stress and heat acclimation and their involvement in host response to heat stress were summarized recently (Wu et al., 2020a). Heat stress, potentially regulated by epigenetic modifications, such as DNA methylation, DNA hydroxymethylation, and histone modifications, was reported to significantly affect bovine embryonic development and fertility (Mendes et al., 2017; de Barros and Paula-Lopes, 2018; Diaz et al., 2019; Sun et al., 2019). Epigenetic regulation was associated with the effects of betaine on heat stress reduction in poultry (Nayak et al., 2016; Saeed et al., 2017). In addition, DNA methylation and histone H3K27me3 and H3K4me3 changes were identified to partly regulate the adaption of chickens to embryonic thermal manipulation, which is crucial for improving their thermal adaptability to heat stress in postnatal life (Kisliouk et al., 2017; Vinoth et al., 2018; David et al., 2019). DMRs and associated genes with roles in energy and lipid metabolism, cellular defense, and stress responses were identified in longissimus dorsi muscles of heat-stressed pigs indicative of roles of epigenetic regulation of pig muscle development, meat quality, and heat stress processes in pigs (Hao et al., 2016). Increased m^6^A RNA methylation level and increased expression levels of m^6^A-related enzymes and heat stress proteins were observed in the liver of sheep after heat stress, indicating involvement of m^6^A in the regulation of host response to heat stress (Lu et al., 2019).

**TABLE 3:** Epigenetic changes impacting livestock health.

Stimulus or disease	Breed	Organ	Epigenetic alteration	References
**Environment stress**				
Heat stress (embryonic thermal manipulation)	Chicken; PB-2 and NN lines	Brain tissue (hypothalamus)	Altered methylation at *HSPs*	Kisliouk et al., 2017; Vinoth et al., 2018
Heat stress (embryonic thermal manipulation)	Broiler chickens	Hypothalamus and muscle tissues	785 H3K4me3 and 148 H3K27me3 differential peaks in the hypothalamus	David et al., 2019
Heat stress	Male DLY pigs	*Longissimus dorsi* muscles	57,147 DMRs corresponding to 1,422 DMGs	Hao et al., 2016
Heat stress	Hu sheep	Liver	Increased m^6^A on RNA of HSPs	Lu et al., 2019
Hypoxic stress (high and low altitude)	Tibetan pigs	*Longissimus dorsi* muscles	DMRs mainly involved in starch and sucrose metabolism	Jin L. et al., 2018
Hypoxic stress (different species and high/low altitude)	Tibetan and Yorkshire pigs	Heart	6,829, 11,997, 2,828, and 1,286 DMRs between TH vs YH, TL vs YL, TH vs TL, and YH vs YL	Zhang et al., 2019
Hypoxic stress (different species and highest plateau)	Tibetan goat, Tibetan sheep, Chuanzhong goat, and small-tailed Han sheep	Various tissues, including the heart, liver, lungs, kidney, muscle, and brain	Higher methylation in *HIF-1*α, *HIF-3*α, and *EPO.* Lower methylation rate in *FIH-1*	Wang et al., 2017g
Prenatal (transportation stress)	Brahman cows and their offspring	Blood	7,407 hyper- and 8,721 hypomethylated CpG sites, including 1,205 DMCs located within promoter regions	Littlejohn et al., 2018
**Disease**				
*Mycobacterium bovis* infection	Holstein–Friesian cattle	CD4 + T cells	Genome-wide DNA methylation profile revealed 760 DMRs	Doherty et al., 2016
*Mycobacterium bovis* infection	*In vitro*	Alveolar macrophages	H3K4me3 is more prevalent in chromatin, at a genome-wide level in the infected group	Hall et al., 2019
Johne’s disease	Holstein cows	Ileum and ileum lymph node	2,000 DMCs and 205 DMRs in the ileum, 6,394 DMCs and 3,946 DMRs in ileum lymph node in response to *Mycobacterium avium* spp. *paratuberculosis*	Ibeagha-Awemu et al., 2020a,b
Clinical mastitis	Holstein cattle	Blood	Altered methylation of *CD4*	Wang et al., 2013; Usman et al., 2016
Clinical mastitis	Holstein cows	Mammary glands	Changed DNA methylation-regulated *IL6R* transcription	Zhang et al., 2018a
*E. coil-* and *S. aureus*-induced mastitis	Holstein cows	Mammary glands, peripheral blood	Genome-wide DNA methylation profile revealed a plethora of DMRs	Song et al., 2016; Sajjanar et al., 2019; Ju et al., 2020; Wang et al., 2020c; Wu et al., 2020b
*E. coil*-induced mastitis	Holstein cows	Lymphocytes	H3K27me3 levels in silent genes were higher in subclinical *S. aureus* mastitis cattle than in healthy cows	He et al., 2016
LPS challenge	Cows	Endometrial cells	Decreased methylation of specific CpG sites at IL-6; increased expression level of *IL-6*, *IL-8*, and *DNMTs*	Wang et al., 2018
LPS challenge	*In vitro*	Mammary epithelial cell	High LPS dose induced hypomethylation of immune-related genes, while low LPS dose induced hypermethylation of lactation-related genes	Chen J. et al., 2019
BVDV	*In vitro*	Madin–Darby bovine kidney cell	*DNMT1* silencing induced decreased methylation level of miR-29b and repressed BVDV replication	Fu et al., 2017
Subacute ruminal acidosis	Dairy goat	Liver	Reduced promoter methylation level of *LOC101896713* and *CASP8*	Chang et al., 2018
Scrapie	Sheep	Thalamus	8,907 DMRs	Hernaiz et al., 2019
Poly I:C stimulation	Dapulian and Landrace pigs	Peripheral blood mononuclear cell	5,827 DMRs and 70 DM and DE genes	Wang et al., 2017c
Bacteria colonization in intestine	Danish Landrace × Large White × Duroc pigs	Distal small intestine	Changed genome-wide DNA methylation related to the expression of immune genes	Pan et al., 2018, 2020
*E. coli* challenge	Sows	Mammary epithelial cells	561 and 898 DMCs at 3 and 24 h after *E. coli* challenge	Sajjanar et al., 2019
Porcine epidemic diarrhea virus	Large White piglets	Jejunum	Higher H3K4me3 enrichment and expression levels of some antiviral genes in the infected group	Wang H. et al., 2019
PRRSV	Transgenic pigs	serum and lung tissues	*HDAC6* overexpression enhances resistance to PRRSV infection	Lu et al., 2017
H5N1 influenza virus	BWEL-SPF chicken	Spleen, thymus, and bursa of Fabricius	Significantly upregulated total DNA methylation levels in the thymus and bursa of the infected group	Zhang Y. et al., 2016
*Salmonella enterica* infection	Domestic chickens	Blood	879 DMRs including 135 DMRs in the promoter regions	Wang et al., 2017a
Marek’s disease	Two specific pathogen-free inbred lines of White Leghorn	Bursa of Fabricius	Different H3K27me3 markers associated with immune-related genes	Mitra et al., 2015; Song, 2016
NDV infection under heat stress condition	Fayoumi and Leghorn	Bursa	Greater differences in histone modification (H3K27ac and H3K4me1) levels in Leghorns than Fayoumis, associated genes enriched in biological processes gene ontology terms related to cell cycle and receptor signaling of lymphocytes	Chanthavixay et al., 2020b

*H3K4me3, trimethylation at the fourth lysine residue of histone H3 protein; H3K27me3, trimethylation at the 27th lysine residue of histone H3 protein; DMR, differential methylated region; DMG, differential methylated gene; HSP, heat-shock protein; DMC, differential methylated CpG; TH, Tibetan pigs grown in the highland; TL, Tibetan pigs grown in the lowland; YH, Yorkshire pigs grown in the highland; YL, Yorkshire pigs grown in the lowland; *E. coil*, *Escherichia coli*; *S. aureus*, *Staphylococcus aureus*; LPS, lipopolysaccharide; BVDV, bovine viral diarrhea virus; PRRSV, porcine reproductive and respiratory syndrome virus; DM, differentially methylated, DE, differentially expressed; NDV, Newcastle disease virus; H3K27ac, acetylation at the 27th lysine residue of histone H3 protein; H3K4me1, the monomethylation at the fourth lysine residue of the histone H3 protein.*

Hypoxic stress is an important environmental stressor affecting porcine growth, especially in high-altitude regions. Genome-wide DNA methylation profiles of porcine tissues from pigs raised in regions of different altitudes revealed important regulatory roles of DNA methylation in porcine hypoxia adaptation (Jin L. et al., 2018; Zhang et al., 2019). For example, some DMGs identified in heart tissues of Tibetan pigs from high- and low-altitude regions were significantly enriched in hypoxia-inducible factor (HIF) 1 signaling pathway suggesting impact on hypoxia-related processes (Zhang et al., 2019). Particularly, DNA methylation mediated the expression of *SIN3A* and *CACNG6* in longissimus dorsi muscle of Tibetan pigs during low-altitude acclimation (Jin L. et al., 2018). Moreover, methylation changes in hypoxia genes, such as higher methylation levels in *HIF-1*α, *HIF-3*α, and *EPO* and lower methylation level in *HIF-1*, were identified in the heart, liver, lungs, kidney, muscle, and brain tissues of plateau goat and sheep, suggesting the involvement of epigenetic regulatory mechanisms of hypoxia resistance of plateau animals (Wang et al., 2017g).

In cattle, maternal stress due to transportation was identified as a potential factor that induced methylome changes in Brahman bull calves, whereby thousands of hyper- and hypomethylated CpG sites were identified compared with non-transported control calves (Littlejohn et al., 2018). The methylome changes were through increased DNA methylation sites at promoter regions of genes enriched in pathways related to behavior, stress response, metabolism, and immune response, which induced the repression of their transcriptional activities (Littlejohn et al., 2018). In goat, lowered global DNA methylation level was thought to be involved in upregulated activity of caspase-3 and caspase-8 enzymes, increased expression of inflammatory cytokines (*IL-10*, *IL-1*β, and *iNOS2*) and activation of TLR-4 and NF-κB pathways in response to chronic stress induced by long-term application of low doses of dexamethasone in colonic epithelium of goats (Cai et al., 2019).

#### Epigenetic Regulation of Livestock Immune Response to Disease Pathogens

Epigenetic modifications are known to significantly affect the dynamic regulation of immune responses to infection and other stressors (Emam et al., 2019; Safi-Stibler and Gabory, 2019). Studies on DNA methylation and the immune response have described the methylation of immune-related genes and the global DNA methylation patterns in response to varied disease pathogens (Table 3). For example, DNA methylation was found to directly affect gene expression in CD4+ T cells during an infection of *Mycobacterium bovis* in cattle (Doherty et al., 2016). The promoter region of miR-29b showed significant decreased methylation level in Madin–Darby bovine kidney (MDBK) cell line infected with bovine viral diarrhea virus (BVDV; Fu et al., 2017). Moreover, silencing of *DNMT1* expression in MDBK significantly decreased miR-29b promoter methylation and upregulated its expression, as well as repressed BVDV replication, supporting the interaction between DNA methylation and miRNA in the regulation of livestock health (Fu et al., 2017). Bacterial LPS stimulation of endometrial cells resulted in increased expression of immune-related genes (*IL-6* and *IL-8*), which was enhanced by the inhibition of DNA methylation (Wang et al., 2018). In bovine mammary epithelial cells, altered methylome (mainly hypermethylation) in response to lower doses of LPS (1–10 EU/ml) impacted the expression of genes (e.g., *ACACA*, *ACSS2*, and *S6K1*) related to milk production (lipid and amino acid metabolism), while high LPS doses (>10 EU/ml) induced hypomethylation of genes in immune response pathways (Chen J. et al., 2019). DNA methylation was reported to regulate the expression of *IL-6R* rather than genetic mutations in response to mastitic pathogen (Zhang et al., 2018a). Moreover, the co-stimulation of bovine mammary epithelial cells with LPS, peptidoglycan (PGN), and lipoteichoic acid (LTA) significantly increased DNA hypomethylation compared with LPS stimulation, indicating that the additive effects of co-stimulation decreased methylation levels resulting in increased transcriptome changes and inflammatory responses (Wu et al., 2020c). The hypermethylation of the *CD4* promoter was reported to repress its gene expression in Holstein cows with clinical mastitis (Wang et al., 2013; Usman et al., 2016). Recently, *NCKAP5* and transposon *MTD* were found to be differentially methylated in a mouse model of mastitis, indicating their potential effects on the development of *Staphylococcus aureus* mastitis and their potential as epigenetic markers of *S. aureus* mastitis (Di Wang et al., 2020). Furthermore, a plethora of DMRs were identified in bovine mammary gland tissues in response to mastitis caused by different pathogens, including *Escherichia coil* and *S. aureus*, revealing crucial regulatory roles of DNA methylation in mammary immunity during mastitis (Sajjanar et al., 2019; Wu et al., 2020b). Moreover, genome-wide DNA methylation alteration in the format of C^m^CGG was significantly related to the immune response to *S. aureus*-induced mastitis, and several genes including *IL-6R*, *TNF*, *BTK*, *IL-1R2*, and *TNFSF8* were identified as potential epigenetic markers of *S. aureus* mastitis (Wang et al., 2020c). DMRs were also identified in peripheral blood from mastitis-infected cattle, further demonstrating the importance of DNA methylation in host immune response (Song et al., 2016; Ju et al., 2020).

In addition to DNA methylation, histone modifications also contribute to mammary gland immunity (Silva et al., 2018; Wu et al., 2020b). For example, inhibition of histone deacetylase increased the expression of β-defensin and possibly improved host resistance to intramammary infections (Kweh et al., 2019). Besides, H3K27me3 in the upstream region of key genes like *IL-10*, *PTX3*, etc. regulated their expression in bovine lymphocytes in response to *S. aureus* mastitis (He et al., 2016). Recently, integration of chromatin immunoprecipitation sequencing (ChIP-seq), RNA sequencing, and miRNA sequencing data from *M. bovis*-infected macrophage revealed that bovine alveolar macrophage transcriptional reprogramming arises through discrepant distribution of H3K4me3 and RNA polymerase II at key immune genes (Hall et al., 2019). However, no differences were found between the methylomes of healthy and *M. bovis*-infected bovine alveolar macrophage 24 h post infection suggesting that DNA methylation may be less involved in the early host response to *M. bovis* (O’Doherty et al., 2019).

In dairy goats, reduced promoter methylation contributed to the regulated expression of key genes related to inflammation and apoptosis in the liver during subacute ruminal acidosis induced by high-concentrate diets (Chang et al., 2018). Additionally, abnormal DNA methylation levels of genes with roles in signaling and transportation and their involvement in the pathogenesis of scrapie were identified in sheep with scrapie compared with healthy controls (Hernaiz et al., 2019). Dynamic DNA methylation changes have also been reported to impact porcine immune responses by regulating the expression of immune-related genes. A high number of DMRs showing inverse association with gene expression were identified in peripheral blood mononuclear cells in response to poly I:C stimulation, as well as 70 differently methylated and expressed genes with related functions in the regulation of the immune system and leukocyte activation (Wang et al., 2017c). Differential gene expression in response to poly I:C and LPS stimulation was also reported to be significantly associated with H3K27ac alteration at active regulatory regions enriched for TF binding motifs of TFs with roles in the inflammation response (Herrera-Uribe et al., 2020). In addition, involvement of DNA methylation in the regulation of the expression of intestinal immune metabolism-related genes during bacteria colonization immediately after birth and the subsequent influence on newborn intestinal immune development has been reported (Pan et al., 2018, 2020). Promoter methylation level of *BPI* gene in Yorkshire, Sutai, and Meishan pigs was negatively associated with its gene expression and contributed to intestinal immunity and disease susceptibility (Wang et al., 2017d). Promoter methylation in *PACSNI1* repressed its expression and indirectly promoted the production of *IL−6*, *IL−8*, and *TNF*α, indicating its potential to mediate porcine response to disease pathogens (Feng et al., 2019). In addition to regulatory roles in porcine immune responses, epigenetic mechanisms, including DNA methylation and histone modifications, were frequently observed to play roles in porcine diseases. *E. coli*-induced DNA methylation alteration in the form of DMCs in porcine mammary epithelial cells was mapped to the regulatory regions of immune-related genes, such as *SDF4*, *SRXN1*, *CSF1*, and *CXCL14* (Sajjanar et al., 2019). A total of 1,885 H3K4me3 associated with 1,723 genes were identified in the jejunum of piglets with porcine epidemic diarrhea virus, revealing a positive correlation between higher H3K4me3 deposition and increased expression levels of some antiviral genes, including *AS1*, *OAS2*, *EFNB2*, and *CKS1B* (Wang H. et al., 2019). The overexpression of *HDAC6* enhanced host resistance to porcine reproductive and respiratory syndrome virus (PRRSV) infection, resulting in repressed PRRSV production *in vitro* and lower viral load in the lung and less clinical symptoms *in vivo* (Lu et al., 2017).

Epigenetic modifications also play important regulatory roles in the immune response of chickens. The whole genome-wide DNA methylation patterns of lungs from two chicken lines differing in genetic resistance to multiple pathogens revealed many immune-related gene ontology terms enriched by genes within DMRs, suggesting DNA methylation as a possible regulatory mechanism underlying the immune response differences (Li et al., 2015). A dynamic unstable chromatin structure with nucleosome-free regions, that intermingled with H3K4me3- and H3K27ac-modified nucleosomes, was identified in the body of some genes participating in the innate immune response of chickens (Jahan et al., 2019). Also, 5hmC was associated with B-cell death during the immune response to infectious bursal disease virus infection in chickens (Ciccone et al., 2017). In addition, DNA methylation, histone modifications, and other epigenetic signatures were reported during the immune response to diverse infectious diseases in chickens. The global DNA methylation level of immune organs, including thymus and bursa, was significantly upregulated in chickens with avian influenza virus infection (Zhang Y. et al., 2016). The blood methylome showed slightly higher methylation levels around the transcription start and termination sites in *Salmonella enterica*-infected chickens than healthy controls, and the differentially methylated peaks in the promoter regions were vastly correlated with immune-related genes (Wang et al., 2017a). Marek’s disease virus induced various temporal chromatin signatures to bursa of Fabricius chickens at different stages of Marek’s disease development, and the differential H3K27me3 was significantly enriched in pathways related to the immune response (Mitra et al., 2015; Song, 2016). The response of two genetically distinct highly inbred layer chicken lines (Leghorns and Fayoumis) to Newcastle disease virus (DNV) infection while under heat stress revealed greater differences in histone modification (H3K27ac and H3K4me1) levels in Leghorns than Fayoumis, and the associated genes were enriched in biological processes gene ontology terms related to cell cycle and receptor signaling of lymphocytes, thereby revealing the possible cellular mechanisms underlying the development of genetic variation in NDV resistance (Chanthavixay et al., 2020a). Furthermore, epigenetic reprogramming in the form of histone trimethylation and acetylation is possibly involved in the regulation of gene expression related to improved innate immune system conditioning following vaccination of laying hens (Kang et al., 2019a,b).

## Application of Epigenetics Data in Livestock Production

### Epigenetics Biomarkers in Health Management

A biomarker is a factor or distinctive property or character that can be measured and evaluated as an indicator or gauge of normal biological and pathological processes. The Food and Agricultural Organization defines a biomarker as any substance, structure, or process which impacts or predicts the incidence of disease or its consequences, and could be quantified (World Health Organization [WHO], 2001). Biomarkers are classified into many specific types, including diagnostic, prognostic, predictive, therapy monitoring, and risk biomarkers (Bock, 2009). For clinical application, biomarkers are expected to be specific, sensitive, and stable and could be validated in abundant samples by different labs (Mishra and Verma, 2010).

According to the properties of biomarkers, an epigenetic biomarker is defined as any epigenetic mark or changed epigenetic mechanism that is measurable in different tissues or body fluids and can delineate a disease condition (detection), predict the outcome of disease (prognostic biomarker) or response to therapy or treatment (predictive biomarker) or a monitor of treatment response (therapy monitoring biomarker), or forecast the risk of future disease development (risk biomarker) (García-Giménez et al., 2016). Since epigenetic markers respond to different types of internal (e.g., maternal environment, etc.) and external environmental cues (e.g., nutrition, management practices, disease pathogens, etc.) as directed by the underlying genetic composition during a lifetime, epigenetic biomarkers may represent the evolution of individual phenotype variations and can contribute to improved disease and production management. In addition, the dynamic changes due to extra- or intraenvironmental cellular conditions and disease progression or evolution in response to environmental factors are one advantage of epigenetic biomarkers when compared with stable (not changing) genetic biomarkers based on gene sequence (García-Giménez et al., 2016). Association of genetic biomarkers to phenotypes is often inconsistent across studies, while epigenetic markers are promising substitutes for the timely diagnosis and monitoring of diseases (Rahat et al., 2020). Furthermore, epigenetic markers being tissue specific reflect the pattern of disease progression (Zeng et al., 2019). Moreover, epigenetic markers, especially methylated DNA and miRNA, have high stability in a variety of samples (e.g., tissues, blood, urine, plasma, milk, etc.) and are stable over a range of conditions. Also, a higher spontaneous epimutation rate (three orders of magnitude) than genetic mutation rate in *Arabidopsis thaliana* has been reported (Schmitz et al., 2011), implying a higher spontaneous mutation rate and availability of more raw materials for genetic improvement due to epimutations than genetic or nucleotide mutations. An epimutation, which is different from DNA mutation, is generally defined as a heritable change in gene activity that is linked to gain or loss of DNA methylation or modifications of chromatin (Oey and Whitelaw, 2014). Epimutations have been further separated into primary (occurs in the absence of DNA sequence change) and secondary (occurs secondary to a DNA mutation in a *cis*- or *trans*-acting factor) categories (Horsthemke, 2006). Moreover, epimutations have been described as constitutional, meaning that they are derived from the germline and consequently should be present in all of the tissues of an individual or somatic (arise in cells in somatic tissues) (Hitchins and Ward, 2009). Evidence of how epimutations induced by endocrine disrupting chemicals impact gene expression, potentially leading to the development of heritable disease conditions in humans have been summarized recently (Lehle and Mccarrey, 2020).

To enable application, biomarkers must be characterized and validated. In farm animals, however, epigenetic research is still at the exploratory level, compared with extensive work in humans and model organisms that has enabled the detection of epigenetic biomarkers and application in various conditions. In humans, epigenome-wide association studies (EWAS) have facilitated the identification of epigenetic biomarkers and their association with phenotype of complex traits, such as human longevity, disease predisposition, diseases, etc. (Abbring et al., 2019; Szymczak et al., 2020). Besides, growing EWAS evidence supports the application of epigenetic biomarkers in human disease diagnosis and treatment (Birney et al., 2016; Carnero-Montoro and Alarcón-Riquelme, 2018; Edris et al., 2019). Various epigenetic biomarkers have been identified for different diseases, such as tumors, colorectal cancer, cardiovascular diseases, etc., revealing their potential use in prognostic, prediction, and even treatment (Kamińska et al., 2019; Soler-Botija et al., 2019; Jung et al., 2020). A DNA methylation assay based on *SEPT9* was the first Food and Drug Administration (FDA)-approved cancer test based on DNA methylation and showed high sensitivity (71.1–95.6%) and specificity (81.5–99%) to colorectal cancer, the leading cause of cancer deaths (Tanić and Beck, 2017). In addition, a *GSTP1* methylation assay based on a hypermethylated CpG island in the promoter of *GSTP1* and frequently reported in tumor tissues from prostate cancer patients is under clinical test to improve the detection sensitivity, specificity, and accuracy of early prostate cancer diagnosis (Martignano et al., 2016; Markou et al., 2017). Recently, the discovery of epigenetic drugs promoted the further development of sensitive epigenetic biomarkers for predicting or dealing with disease evaluation (Sistare and DeGeorge, 2007). For instance, DNMT inhibitors, including 5-azacytidine and 5-aza-2’-deoxycytidine, were approved by the FDA and demonstrated to be highly efficient in the treatment of hematological malignancies (Kantarjian et al., 2012; Adès et al., 2013). DNMT inhibitors (azacitidine and decitabine) were reported to significantly improve the survival of patients with myelodysplastic syndromes; however, only about 50% of patients showed good clinical responses that were measurable or visible after 4–6 months of treatment (Lee et al., 2013). To deal with the silent 4–6-month stage, predictive epigenetic biomarkers would have great clinical value to reduce the possible effects of ineffective treatments that may cause side effects, unnecessary cost, and time wastage (Treppendahl et al., 2016).

As discussed in the sections above, diverse alterations of epigenetic markers have been revealed to be significantly associated with livestock health, suggesting their potential as epigenetic biomarkers that could be used for diagnostic, prognostic, predictive, or therapy monitoring. Moreover, environmental factors, such as living or farm environment, feed quality/quantity, pathogens, parental stress, environmental stress, chemicals, etc., directly affect livestock productivity, and these effects captured through epigenetic markers can be included in animal health management. For example, dynamic alterations of epigenetic mechanisms in response to parental nourishment and environmental factors or perturbations, especially at the stage of embryo development during pregnancy, have been demonstrated (Dean et al., 2005; Luo et al., 2018), and their identification and consideration during critical stages of offspring development could lead to healthier pregnancies by including them in farm management strategies. In addition, the identification of possible epigenetic biomarkers underlying these effects could contribute to the evaluation of health and productivity of offspring early in life paving the way for early intervention. Epigenetics biomarkers could be particularly suitable for the detection and management of chronic, silent (no obvious clinical symptoms) livestock diseases, such as metabolic disorders, porcine muscular degenerative disease, chronic mastitis, subacute ruminal acidosis, and paratuberculosis.

### Epigenetic Biomarkers for Breeding Purposes

The contribution of epigenetic modifications to livestock phenotype variation, supported by growing evidence, is gaining importance and supports the potential application of epigenetic biomarkers, especially DNA methylation in livestock breeding programs (González-Recio et al., 2015; Ibeagha-Awemu and Zhao, 2015; Triantaphyllopoulos et al., 2016; Ibeagha-Awemu and Khatib, 2017; Paiva et al., 2019). The potential usefulness of epigenetic biomarkers in livestock breeding is further emphasized by the fact that phenotypic expression is not only a reflection of an individual’s DNA composition or sequence but also a reflection of how the genome is copied and regulated by the epigenome taking into account both past and present environmental influences or information (Ibeagha-Awemu and Khatib, 2017). Furthermore, epigenetic inheritance (also known as non-genetic inheritance or transgenerational epigenetic effects) refers to any modification in offspring phenotype that is due to the transmission of factors other than DNA sequence information from parents or ancestors (Bonduriansky and Day, 2009). Epigenetic inheritance has been reported to play crucial roles in phenotypic variation during one’s own and offspring development (Triantaphyllopoulos et al., 2016; Nilsson et al., 2018). Therefore, epigenetic inheritance, including intragenerational and transgenerational inheritance, underscores the notion that individual phenotype modifications could at least partly result from the environmental effects on founder generations during key developmental stages of germline cells (Skinner, 2011; Nilsson et al., 2018; Skinner et al., 2018). Therefore, the transmission of epigenetic biomarkers, such as DNA methylation, histone modifications, and ncRNAs, between generations plays a part in epigenetic inheritance in livestock animals (Feeney et al., 2014; Triantaphyllopoulos et al., 2016; Thompson et al., 2020). The current genetic data used for livestock breeding could only explain a portion of phenotypic variance or trait heritability, and supplementing genetic data with epigenetic biomarkers could improve the prediction accuracy of breeding values (González-Recio et al., 2015; Ibeagha-Awemu and Khatib, 2017; Yakovlev, 2018).

As discussed in the sections above, epigenetic variations ranging from single sites to epigenome-wide maps and their regulatory mechanisms of gene expression have been reported in different tissues of several mammalian species (Ibeagha-Awemu and Zhao, 2015; Chavatte-Palmer et al., 2018; Ahmad et al., 2019; Morales-Nebreda et al., 2019; Choi et al., 2020). Furthermore, association between single nucleotide polymorphisms (SNPs) and differential DNA methylation has been reported, indicating that methylation alteration leads to variable expression of related genes and thereby phenotype determination (Banovich et al., 2014; Imgenberg-Kreuz et al., 2018). The alteration of CpG sites caused by SNP suggested one possible mechanism that SNP impacts gene expression by the altered epigenetic patterns, thereby suggesting the possible application of epigenetic biomarkers in livestock improvement breeding (Zhi et al., 2013; Maldonado et al., 2019). However, data to support the exploration and application of epigenetic biomarkers in livestock breeding is currently limited. For instance, data on the contribution of epigenetic alterations to the heritability of livestock health and production traits are not available. Moreover, statistical methods are urgently needed to support quantification of the exact contribution of epigenetic biomarkers to phenotype variation. It was proposed recently that the effects of genetic and non-genetic inheritance should be dissected and considered in the estimation of trait heritability (Danchin et al., 2011). Therefore, the animal or mixed-effects models or statistical approaches that support the simultaneous evaluation of several variance components can be expanded to include the non-genetic or epigenetic components of variation for the purpose of livestock breeding (Tal et al., 2010; Ibeagha-Awemu and Khatib, 2017). Thence, the relationship between DNA methylation biomarkers with production traits, identified through EWAS, could be included in the development of new breeding methods to enable quantification of the epigenetic contribution to the prediction of breeding values.

## Research Gaps and Future Perspectives

### Develop Tools for Livestock Epigenetic Research

Next-generation sequencing methods, such as WGBS, reduced representation bisulfite sequencing (RRBS), ChIP-Seq, etc., have supported the profiling of epigenetic markers at a genome-wide scale in livestock species. However, only a limited number of samples can be profiled at a time due to the cost associated with using these technologies. Furthermore, data generated on a limited number of samples is not adequate for use in improvement management/breeding. Therefore, less expensive tools that support application in a large number of samples are needed to support the application of epigenetic information in livestock production. In humans for example, array-based DNA methylation arrays have been developed to support EWAS and for further application in disease diagnosis and treatment (Birney et al., 2016; Carnero-Montoro and Alarcón-Riquelme, 2018). The Infinium^®^ HumanMethylation450 BeadChip methylation array (450K) is popularly used to detect methylation changes at 450,000 CpG sites in the human epigenome, and was recently updated to Infinium MethylationEPIC BeadChip array (850K) with doubled coverage (over 850,000 CpG sites) of methylation sites (Sandoval et al., 2011; Moran et al., 2016). The high accuracy and reliability of DNA methylation measurement and association with biological traits, in hundreds to thousands of samples based on arrays, promoted the wide application of DNA methylation in EWAS in humans (Li M. et al., 2019). The lack of commercially available epigenome analysis assays severely restricts the application of EWAS for uncovering the epigenetic biomarkers associated with livestock health and production traits. The development of epigenome-wide arrays for epigenetic pattern identification in large samples becomes a prerequisite for the application of epigenetic biomarkers in livestock breeding and production management. Therefore, there is an urgent need for the development of livestock-specific assays based on epigenetic mechanisms (especially DNA methylation) with high reliability and commercial availability. Besides, livestock epigenetics research is developing with the potential to improve the reliability and accuracy of breeding values estimation with possible application in livestock management, breeding, and selection.

Genome editing technologies which have been successfully used to modify livestock phenotype through the introduction of useful alleles for heat tolerance, disease resistance (e.g., tuberculosis, mastitis, bovine respiratory disease), production (e.g., production of male-only offspring, myostatin gene knockout), elimination of allergens (e.g., beta-lactoglobulin gene knockout), and welfare (e.g., introduction of polled or hornlessness) into livestock populations [reviewed by Mueller et al. (2019); Van Eenennaam (2019), and Bishop and Van Eenennaam (2020)] hold great promise for furthering the application of epigenetic modifications in livestock improvement. Moreover, application of epigenetic editing at specific loci of interest epitomizes an innovative procedure that might selectively and heritably alter gene expression (Vojta et al., 2016).

### Expand Epigenetic Exploration in Livestock Organs and Tissues Under Varying Conditions

As demonstrated in the sections above, epigenetic markers contribute to livestock phenotype variation. Moreover, the epigenome responds to the exposome (nutrition, pathogens, chemicals, maternal behavior, parental environment, climatic conditions, environment, management practices, etc.) in a tissue- and cell-specific manner. With current advances in sequencing and data management technologies, the possibility for genome-wide analysis of epigenetic modifications in specific livestock tissues in response to the exposome is enormous. However, compared with human and model organisms, epigenetic studies in livestock is less developed. Many possible reasons have been advanced to explain this, including limited available funding, research tools and epigenetic research activities on livestock, insufficient recognition of the contribution of epigenetic variation to livestock phenotype diversity, and limited involvement of a considerable number of research professionals in livestock epigenetic research (Ibeagha-Awemu and Zhao, 2015). Efforts of the international consortium on functional annotation of animal genomes (FAANG Project, www.faang.org) and various genome-wide DNA methylation or histone modifications profiling (mentioned in the sections above) have reported epigenetic variation in diverse but limited tissues of livestock species (Foissac et al., 2019; Halstead et al., 2020). Therefore, more efforts are needed to explore the epigenetic variations and biomarkers in different livestock organs/tissues under varying conditions and their contribution to phenotypic expression. Furthermore, monitoring of the dynamic changes of epigenetic markers in response to environmental factors, such as nutritional changes and diseases, could be used to develop new health and disease detection and prediction tools.

### Recognize Epigenetic Contribution to Livestock Phenotype Diversity

Even with mounting evidence supporting the contribution of epigenetic modifications to livestock phenotype variation, there is limited recognition and exploitation of the contribution of epigenetic biomarkers to phenotype variation in livestock management and breeding. Development of advanced statistical methods is required to enhance the understanding of how epigenetic markers interact with genetic factors to influence phenotype diversity of production and health traits in livestock. Furthermore, potential epigenetic regulation has been explored only in a handful of traits and conditions. Moreover, most recent investigations paid attention to the epigenetic modifications in response to single factors, but majority of livestock traits are modulated by the interaction of multiple factors, therefore deserving holistic approaches. Therefore, identification of the epigenetic contributions to livestock traits under the influence of multiple factors is needed and will bring added value for the utilization of epigenetic biomarkers in livestock management. Moreover, limited studies have focused on the linkage between epigenetic modifications and developmental outcomes. Thus, in-depth exploration linking epigenetics and related physiologic responses may further our understanding of the mechanisms underlying livestock productivity and health.

### Examine, Document, and Exploit Epigenetic Inheritance in Livestock

Among the epigenetic studies carried out in livestock, a limited number focused on epigenetic inherence and epigenetic transgenerational biomarkers detection (Triantaphyllopoulos et al., 2016). A major challenge in the examination of epigenetic inherence and its potential application in livestock breeding is the ability to trace epigenetic variations between generations. Accumulating evidence indicates that environmentally induced epigenetic biomarkers could be acquired and used to form transgenerational memory that is partly responsible for environmentally induced heritable traits (Heijmans et al., 2008; Daxinger and Whitelaw, 2010). As summarized in Tables 1–3, many factors, such as pathogens, nutrition, etc., are associated with epigenetic alterations during individual development, especially in germline cells. It has been reported that environmentally induced epigenetic transgenerational inherence of DNA methylation changes in sperm promoted genome instability such as changes of copy number variations in next generations (Skinner et al., 2015). However, the effects of environmental variables on offspring still cannot be fully and directly estimated. The stochastic changes of epigenetic biomarkers act as potential intermedium between environmental variables and related phenotypic variation. In addition, stochastic epigenetic changes can generate tissue- or cell-specific epigenetic variability over time without changes in DNA sequence, contributing to explain the phenotypic variation that cannot be explained by genetic mechanisms (Petronis, 2010). Therefore, recognition of the significance of transgenerational epigenetic inheritance for animal breeding purposes will also facilitate further exploration of currently identified epigenetic effects and their applications in livestock production (Feeney et al., 2014).

### Explore Potential Applications of Epigenetic Biomarkers in Livestock Production and Health

With the development of livestock epigenetic research, reliable epigenetic biomarkers related to productivity and health could be identified and used in livestock management (Franco, 2017; Lin et al., 2019). For example, epigenetic biomarkers in embryo biopsies, placenta, or newborn blood could be discovered and used to develop predictive biomarkers for future phenotypes of interest later in life. Moreover, epigenetic biomarkers in sperm could be used for the selection of sires and sperm quality. Possible monitoring of dynamic epigenetic biomarkers during individual development has potential for use to predict responses to environmental exposure and stressors before observable phenotypic changes. Epigenetic biomarkers could be used to improve the efficiency of different diets, disease diagnosis, and treatments and determine cost-saving avenues (time and money) for precision livestock management.

## Conclusion

This review summarized recent epigenetic reports in livestock and discussed the potential application of epigenetic processes in livestock productivity and health management. The wealth of epigenetic modification data constantly being discovered in livestock has the potential to contribute to enhanced livestock productivity and health. A better understanding of epigenetic modifications, such as DNA methylation, is expected to compliment information on genome processes, including molecular, cellular, biological, and immune responses, and provide deeper insights on how they interact to define phenotypic outcome. Given the high dependence of humans on foods of animal origin and the need to protect the environment, information on epigenetic regulatory processes has the potential to support the development of strategies for increased productivity of livestock animals with minimal environmental impacts. With regard to livestock reproduction, development, growth, productivity, product quality, health, and the immune response, the role of epigenetics and the underlying mechanisms remains to be fully clarified. Knowledge of epigenetic impacts on livestock health can potentially support the development of strategies to lower disease incidence and increase disease resistance in livestock. It also can increase the suitability and efficiency of diagnostic measures, control approaches, such as vaccination, and treatments. To promote the successful application of epigenetics information in livestock management and improvement, more studies and tools are needed to examine the epigenetic effects and to develop strategies for implementation. However, the current comprehension and exploration of epigenetic mechanisms and their potentials in livestock health and production management is far from complete. More studies are therefore needed to get a better understanding of the epigenetic mechanisms underlying phenotypic variation in livestock production and health.

## Author Contributions

EI-A conceptualized the study and obtained funding. MW and EI-A made substantial, direct, and intellectual contribution to the work and approved it for publication.

## Conflict of Interest

The authors declare that the research was conducted in the absence of any commercial or financial relationships that could be construed as a potential conflict of interest.

## Abbreviations

5hmC: 5-hydroxymethylcytosine
5mC: 5-methylcytosine
*ABCA1*, subfamily A: member 1
*ABCA7*, subfamily A: member 7
*ABCG1*: ATP binding cassette subfamily G member 1
*ACACA*: acetyl-coenzyme A carboxylase alpha
*ACC*: acetyl-CoA carboxylase
*ACOX2*: acyl-CoA oxidase 2
*ACSL1*: acyl-CoA synthetase long chain family member 1
*ACSS2*: acyl-CoA synthetase short-chain family member 2
*ADAMTS3*: ADAM metallopeptidase with thrombospondin type 1 motif 3
*Akt*: serine/threonine protein kinase Akt
ATAC: Ada-Two-A-Containing complex
BAF: BRM/BRG1-associated factor complexes
*BTK*: Bruton tyrosine kinase
*BPI*: bactericidal/permeability-increasing protein
BVDV: bovine viral diarrhea virus
*CACNA1S*: calcium voltage-gated channel subunit alpha1 S
*CACNG6*: calcium voltage-gated channel auxiliary subunit gamma 6
*CAPN10*: calpain 10
*LOC101896713*: caspase-3
*CASP8*: caspase-8
*CAV1*: caveolin-1
*CD4*: CD4 molecule
*CKS1B*: CDC28 protein kinase regulatory subunit 1B
*COL6A1*: collagen type VI alpha 1 chain
CpG: cytosine-phosphate-guanosine
*CRYL1*: crystallin lambda 1
*CSF1*: colony stimulating factor 1
*CSN1S1*: alpha s1 casein
*CTCF*: CCCTC-binding factor
*CXCL14*: C-X -C motif chemokine ligand 14
*DICER1*, dicer 1: ribonuclease III
*DLK1*: delta like non-canonical Notch ligand 1
DMC: differentially methylated cytosines
DMG: differentially methylated gene
DMR: differentially methylated region
*DNMTs*: DNA methyltransferases
*DNMT1*: DNA methyltransferase 1
*DNMT2*: DNA methyltransferase 2
*DNMT3A*: DNA methyltransferase 3 A
*DNMT3B*: DNA methyltransferase 3 B
*DNMT3C*: DNA methyltransferase 3 C
*DNMT3L*: DNA methyltransferase 3 L
*E. coli*: *Escherichia coli*
*E2F2*: E2F transcription factor 2
*EEF1D*: eukaryotic translation elongation factor 1 delta
*EFNB2*: ephrin B2
*EGFR*: epidermal growth factor receptor
*EHMT2*: euchromatic histone lysine methyltransferase 2
*ElF5*: E74-like factor 5
*ENPP3*: ectonucleotide pyrophosphatase/phosphodiesterase 3
*EPO*: erythropoietin
*ER* α: estrogen receptor 1 (alpha)
EWAS: epigenome-wide association studies
*FADS6*: fatty acid desaturase 6
*FASN*: fatty acid synthase
*FAT-1*: FAT atypical cadherin 1
*FATP1*: fatty acid transport protein 1
*FDFT1*: farnesyl-diphosphate farnesyltransferase 1
*FTO*: fat mass- and obesity-associated protein
*GALK1*: galactokinase 1
*GLUT1*: glucose transporter 1
*GR*: glucocorticoid receptor
*GSTP1*: glutathione S-transferase Pi 1
*GSTT1L*: glutathione S-transferase theta 1-like
GWAS: genomic-wide association studies
H3K27ac: the acetylation at the 27th lysine residue of the histone H3 protein
H3K26me3: the trimethylation at the 26th lysine residue of the histone H3 protein
H3K27me3: the trimethylation at the 27th lysine residue of the histone H3 protein
H3K36me3: the trimethylation at the 36th lysine residue of the histone H3 protein
H3K4me1: the monomethylation at the fourth lysine residue of the histone H3 protein
H3K4me3: the trimethylation at the fourth lysine residue of the histone H3 protein
H4K12ac: the acetylation at the 12th lysine residue of the histone H4 protein
*HDAC2*: histone deacetylase 2
*HDAC3*: histone deacetylase 3
*HDAC6*: histone deacetylase 6
*HIF-1* α: hypoxia-inducible factor 1 subunit alpha
*HIF-3* α: hypoxia-inducible factor 3 subunit alpha
*HOTAIR*: HOX transcript antisense RNA
*HOXC8*: homeobox C8
*HP*: haptoglobin
*HSD17B2*: hydroxysteroid 17-beta dehydrogenase 2
*IDH2*: isocitrate dehydrogenase (NADP(+)) 2
IGF1: insulin-like growth factor 1
*IGF2*: insulin-like growth factor 2
*IGF2R*: insulin-like growth factor 2 receptor
*IGFBP4*: insulin-like growth factor binding protein 4
*IL-10*: interleukin-10
*IL-1* β: interleukin-1 beta
*IL − 6*: interleukin − 6
*IL-6R*: interleukin-6 receptor
*IL − 8*: interleukin − 8
*IL-1R2*: interleukin 1 receptor type 2
*iNOS2*: inducible nitric oxide synthase 2
*INS*: insulin
*KAT2A*: lysine acetyltransferase 2A
Kcnq1: potassium voltage-gated channel subfamily Q member 1
*KCNQ3*: potassium voltage-gated channel subfamily Q member 3
*LBP*: lipopolysaccharide binding protein
LPS: bacterial lipopolysaccharide
*MAPRE1*: microtubule associated protein RP/EB family member 1
MBD: methyl-binding domain
*MBD1*: methyl binding domain 1
MDBK: Madin–Darby bovine kidney
MeCP1: methyl-CpG binding protein 1
MeCP2: methyl-CpG binding protein 2
Met: methionine
*MTTP*: microsomal triglyceride transfer protein
*MUSTN1*, musculoskeletal: embryonic nuclear protein 1
*MYB*: myb proto-oncogene protein
*NCKAP5*: NCK-associated protein 5
ncRNA: non-coding RNA
NDV: Newcastle disease virus
*NPY*: neuropeptide Y
*NR4A1*: nuclear receptor subfamily 4 group A member 1
*NRF1*: nuclear respiratory factor 1
*NTN1*: netrin 1
NuRD: nucleosome remodeling and deacetylation complex
*OAS1*: 2 ′ –5 ′ -oligoadenylate synthetase 1
*OAS2*: 2 ′ –5 ′ -oligoadenylate synthetase 2
*PACSNI1*: protein kinase C and casein kinase substrate in neurons 1
*PCK1*: phosphoenolpyruvate carboxykinase 1
*PEMT*: phosphatidylethanolamine N-methyltransferase
PHF14: PHD Finger Protein 14
*PITX1*: paired-like homeodomain transcription factor 1
*POMC*: proopiomelanocortin
*PPARG2*: peroxisome proliferator-activated receptor gamma
*PPAR* α: peroxisome proliferator-activated receptor alpha
*PPAR* δ: peroxisome proliferator-activated receptor delta
PPAR γ: peroxisome proliferator-activated receptor gamma
PRRSV: porcine reproductive and respiratory syndrome virus
*PTX3*: pentraxin 3RRBS: reduced representation bisulfite sequencing
RSC: remodel the structure of chromatin
*RXR* α: retinoid X receptor alpha
*RYR1*: ryanodine receptor 1
*S6K1*: ribosomal protein S6 kinase 1
*S. aureus*: *Staphylococcus aureus*
*SAA3*: serum amyloid A 3
*SALL4*: spalt-like transcription factor 4
*SCD1*: stearoyl-CoA desaturase 1
*SDF4*: stromal cell derived factor 4
*SEPT9*: Septin 9
*SIN3A*: SIN3 transcription regulator family member A
*SIRT2*: sirtuin 2
*SIRT4*: sirtuin 4
*SIX1*: SIX homeobox 1
SNP: single nucleotide polymorphisms
*SNX25*: sorting nexin 25
*SREBF1*: sterol regulatory element binding transcription factor 1
*SREBP1*: sterol regulatory element binding protein 1
*SRXN1*: sulfiredoxin 1
*SS18*: SS18 subunit of BAF chromatin remodeling complex
SWI/SNF: switch/sucrose non-fermentable complexes
*TCF7L2*: type 2 diabetes gene
TET: ten-eleven translocation methylcytosine dioxygenases
*TGFB3*: transforming growth factor beta 3
*TLR-4*: toll-like receptor 4
*TMEM8C*: transmembrane protein 8c
*TNF*: tumor necrosis factor α
*TNF*: tumor necrosis factor
*TNFSF8*: TNF superfamily member 8
TSS: transcriptional start sites
*UCP3*: uncoupling protein 3
VTGII: vitellogenin 2
WGBS: whole-genome bisulfite sequencing
WHO: United Nations World Health Organization.
